# Association between hobby engagement and frailty among adults aged 65 years and older in 15 countries: findings from four prospective cohort studies

**DOI:** 10.7189/jogh.16.04208

**Published:** 2026-07-24

**Authors:** Ting Fu, Yuhan Zhao, Yuan Luo, Huiying Wang, Lanzhu Su, Yiqun Miao, Jiawei Li, Yi Qin, Rongrong Guo

**Affiliations:** 1 Department of Critical Care Medicine, Affiliated Hospital of Nantong University, Nantong, China; 2 School of Nursing, Capital Medical University, Beijing, China; 3 Nursing Department, Beijing Gobroad Hospital, Beijing, China; 4 Nursing Department, Affiliated Hospital of Nantong University, Nantong, China

**Keywords:** hobby engagement, frequency, frailty, association, individual participant data meta-analysis

## Abstract

**Background:**

While frailty is a major public health concern in ageing populations, evidence on its relationship with hobby engagement among older adults remains limited. Existing studies have largely been limited to single-country settings and specific hobby types, with little evidence on cross-national generalisability or whether engagement frequency is associated with additional benefit.

**Methods:**

We conducted secondary analyses of harmonised longitudinal data from four nationally representative ageing cohorts conducted across 15 countries between 2010 and 2023: the China Health and Retirement Longitudinal Study, the English Longitudinal Study of Ageing, the US Health and Retirement Study, and the Survey of Health, Ageing and Retirement in Europe. We included participants aged ≥65 with baseline data on hobby engagement, frailty, and covariates, and who had frailty assessed at follow-up using the frailty index (FI). We used linear mixed models and random-effects meta-analyses to estimate the association between hobby engagement and FI, with average marginal effects (AMEs) and their 95% confidence intervals (CIs) reported. We further examined whether this association varied across subpopulations and whether higher engagement frequency was associated with additional benefits, and performed sensitivity analyses to assess the robustness of our findings.

**Results:**

Overall, 43,346 participants from 15 countries were included in the binary hobby-engagement analysis. The hobby engagement frequency analysis included 31,538 participants from 14 countries, as harmonisable information on engagement frequency was unavailable in ELSA. Hobby engagement was associated with lower FI (pooled AME = − 0.044; 95% CI = −0.054, −0.034; *P* < 0.001). Similar associations were consistently observed across age, education, smoking history, alcohol drinking, and physical activity levels, with AMEs from −0.032 to −0.058 and *P*-values <0.05, with no statistically significant interactions detected. Analyses by frequency suggested that hobby engagement at least monthly was associated with a significantly lower FI compared with less than once per month (pooled AME = 0.003; 95% CI = −0.006, −0.001; *P* = 0.028), but higher frequencies, either weekly (*P* > 0.05) or daily (*P* > 0.05), did not provide any additional benefits. Sensitivity analyses confirmed the robustness of our findings.

**Conclusions:**

Hobby engagement was associated with lower subsequent FI among adults aged ≥65 years. The association appeared to plateau beyond a modest engagement level, suggesting that hobby engagement at an appropriate frequency may represent a generalisable and low-cost behavior associated with frailty risk.

Frailty is a common geriatric syndrome characterised by reduced physiological reserve and increased vulnerability to stressors [1]. It is associated with adverse health outcomes, including disability, cardiovascular events, hospitalisation, falls, and even premature death [2]. Its prevalence among community-dwelling adults rises steeply with age, from approximately 11% among adults aged 50–59 years to about 51% in those aged 90 years or older [3]. The growing burden of frailty places substantial strain on individuals, families, and healthcare systems [4]. As the condition is dynamic and potentially reversible, identifying scalable and accessible factors to delay its progression is a public health priority.

Frailty prevention has largely focused on nutrition, exercise, medication management, multidisciplinary intervention, and hormonal supplementation [5], while psychosocial and leisure activities have received less attention, despite their potential accessibility and low cost [6]. Hobbies, such as arts and crafts, reading, volunteering, and club participation, may stimulate cognition and creativity, provide sensory engagement, and support self-expression [7]. From the perspective of the deficit accumulation model, hobby engagement may influence frailty through psychological, biological, social, and behavioural pathways by improving mental well-being, social engagement, and healthier behaviours [8].

However, the current literature remains insufficient to support robust conclusions regarding their association. First, most studies are confined to single-country settings, limiting comparability and overlooking cross-national differences in the social and cultural contexts that may influence both participation patterns and their association with frailty [9]. Second, prior research has typically examined specific hobby types and has not considered hobby engagement more broadly [10]. This may neglect the shared ‘active ingredients’ and underlying mechanisms across different hobbies. Third, it remains unclear whether the association differ by age, sex, health behaviours, socioeconomic position, or residence area. Finally, most studies have treated hobby engagement as a binary exposure, with little consideration of engagement frequency [11], which may help clarify whether the association follows a dose-response pattern or whether a lower threshold of engagement is sufficient to confer benefit, which has direct implications for public health recommendations.

To address these gaps, we harmonised longitudinal data from four population-based ageing cohorts: the China Health and Retirement Longitudinal Study (CHARLS), the English Longitudinal Study of Ageing (ELSA), the US Health and Retirement Study (HRS), and the Survey of Health, Ageing and Retirement in Europe (SHARE). Using a two-stage individual participant data meta-analysis, we aimed to quantify the association between hobby engagement and frailty among adults aged ≥65 years; examine whether this association varies across subpopulations; and investigate whether greater hobby engagement frequency confers additional benefit.

## METHODS

As this study involved secondary analyses of data from four large publicly accessible ageing cohorts, we confirm its adherence to the Journal of Global Health’s Guidelines for Reporting Analyses of Big Data Repositories Open to the Public [12]. (Table S1 in the **Online Supplementary Document**).

### Study design and participants

The CHARLS, ELSA; HRS, and SHARE cohorts used broadly similar study protocols, which are described in detail elsewhere [13–16]. To ensure availability and comparability of hobby engagement and frailty measures, we analysed data over comparable time spans: 2011–2019 for the CHARLS (waves 1–4), 2010–2021 for the ELSA (waves 5–10), 2010–2023 for the HRS (waves 10–16), and 2011–2022 for the SHARE (waves 4–9). Because some SHARE countries did not participate in all survey waves, we restricted its analysis to 12 countries: Austria, Belgium, the Czech Republic, Denmark, Estonia, France, Germany, Italy, Slovenia, Spain, Sweden, and Switzerland. Together with China, England, and the USA from the CHARLS, ELSA, and HRS, respectively, our study covered 15 countries in total (Table S2 in the **Online Supplementary Document**). We only considered participants aged ≥65 years for inclusion, and excluded participants lacking baseline data on hobby engagement or covariates; individuals without frailty measures at baseline; and participants not reporting frailty at any follow-up assessments.

### Assessment of frailty

Using harmonised variables available in the four cohort datasets, we constructed a 30-item frailty index (FI) according to the deficit accumulation approach proposed by Rockwood [17]. The FI encompassed chronic conditions, symptoms, disability, physical function, depression, and cognition (Table S3 in the **Online Supplementary Document**). Items 1–29 were coded as 1 (presence of the deficit) or 0 (absence of the deficit) according to predefined cut-offs, while item 30 (cognitive score) was coded as 1 if a participant’s performance score was at least 1.5 standard deviations below the cohort-specific mean in at least two of the three cognitive tests, and 0 otherwise. We then calculate the FI as the sum of deficits divided by 30, yielding a score from 0 to 1, with higher scores indicating greater frailty [18]. For descriptive purposes and sensitivity analyses, participants were further categorised as robust (FI ≤ 0.10), pre-frail (0.10 < FI < 0.25), or frail (FI ≥ 0.25) [19].

### Determination of hobby engagement

We harmonised hobby engagement using self-reported participation in leisure or social activities. In the CHARLS and the SHARE, hobby engagement was defined as taking part in activities such as volunteering, attending educational or training courses, joining social or sport clubs, playing games (*e.g.* mah-jong, chess, or cards), or reading books and newspapers [16]. In the ELSA, participants were asked whether they had a hobby or pastime [14], while in the HRS, respondents reported how often they worked on a hobby or project [13]. Based on these definitions, we coded hobby engagement as a binary variable (yes/no) and harmonised its frequency into three categories: less than once per week (further subdivided into less than once per month and at least once per month), at least once per week, and almost daily (Tables S4 and S5 in the **Online Supplementary Document**).

### Collection of covariates

Informed by the deficit accumulation model and related evidence [17], we identified eleven baseline variables as covariates, split into demographic characteristics ( age, sex, marital status, residence area, and co-residence with children) [5], socioeconomic variables (education, household wealth, and labour force status) [20], and health behaviours (smoking history, alcohol drinking history, moderate to vigorous physical activity) [21–23].

### Statistical analysis

To minimise potential bias and statistical efficiency loss from excluding participants with incomplete data, we performed multiple imputation by chained equations under the missing-at-random assumption, and pooled estimates using Rubin’s rules [24]. Baseline characteristics by country were presented as means with standard deviations or medians with interquartile ranges (IQRs) for continuous variables or frequencies and percentages for categorical variables.

We used a two-stage individual participant data meta-analysis to pool country-specific estimates [25] (Methods S1 in the **Online Supplementary Document**). In the first stage, linear mixed models were fitted within each country, with FI as a continuous outcome and repeated measurements nested within individuals. Fixed effects included hobby engagement, months since baseline, their interaction, and relevant covariates. Participant-specific random intercepts were included to account for within-person correlation due to repeated measurements. Random slopes for time were tested, but did not improve model fit (chi-squared = 2.45, df = 1, *P* = 0.12) and were therefore excluded. Model assumptions were evaluated using residuals for normality and homoscedasticity. Average marginal effects (AMEs) and their confidence intervals (CIs) were estimated to quantify the adjusted mean change in the FI associated with hobby engagement [26]. We also examined the association between frequency and subsequent FI.

In the second stage, we pooled country-specific AMEs using random-effect meta-analyses. Between-study heterogeneity was quantified as *τ*^2^ using the restricted maximum likelihood method, together with *I*^2^, *H*^2^, and Cochran’s *Q* (*I*^2^ >50% or *H*^2^ >1 indicating substantial heterogeneity) [27]. Meta-analyses were weighted to give greater weight to larger and more precise studies. To explore potential sources of heterogeneity, we conducted meta-regressions with six country-level factors (gross domestic product per capita, Gini index, healthy life expectancy at birth, world happiness index, digital skills score, and Internet use rate) entered separately as independent variables.

Considering that the association between hobby engagement and the FI may differ across subpopulations, we performed similar individual participant data meta-analyses in subpopulations stratified by age group, sex, marital status, education, residence area, smoking history, alcohol drinking history, moderate to vigorous physical activity, co-residence with children, labour force status, and household wealth. Consistent with previous studies, age was grouped as 65–74 (young-old), 75–84 (middle-old), and ≥85 years (oldest-old) [28]. To account for multiple testing, *P*-values for interaction were adjusted using the Benjamini–Hochberg false discovery rate procedure.

To evaluate the robustness of our findings, we performed seven sensitivity analyses. First, we repeated the main analyses using complete cases to address potential bias from missing data. Second, we reran all analyses in participants aged ≥60 to test the robustness of the initial age threshold. Third, we applied inverse probability weights of attrition in linear mixed models to mitigate selection bias due to attrition [29]. Fourth, to minimise bias from reverse causation and inadvertent adjustment for mediators, we repeated the analyses using lagged models in which FI at each follow-up was related to hobby engagement and covariates measured at the immediately preceding wave [30]. Fifth, to address measurement heterogeneity, we reanalysed our data after excluding the ELSA and the HRS data, as hobby engagement was measured differently in these cohorts and neither provided specific hobby examples. Sixth, we repeated analyses after excluding participants from China, given its distinct socioeconomic context as a developing country. Finally, we used Cox proportional hazards models to examine the association between hobby engagement and incident frailty with hazard ratios (HRs) and 95% CIs reported after excluding frail participants at baseline.

We cleaned data in Stata, version SE 15 (StataCorp LLC., College Station, Texas, USA) and performed analyses in *R*, version 4.2.1 (R Core Team, Vienna, Austria). A two-sided *P* < 0.05 indicated statistical significance.

## RESULTS

**Figure 1:**
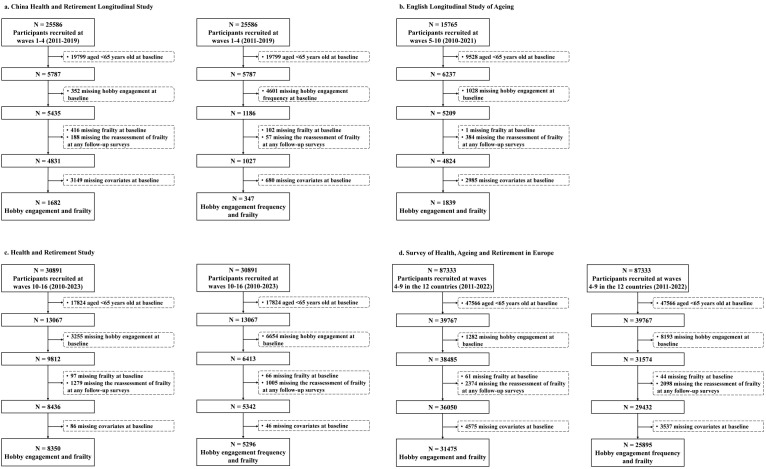
Flowchart of sample selection. **Panel A.** China Health and Retirement Longitudinal Study. **Panel B.** English Longitudinal Study on Ageing. **Panel C.** Health and Retirement Study. **Panel D.** Survey of Health, Ageing, and Retirement in Europe.

We established two analytic samples (**Figure 1**; Tables S6–9 in the **Online Supplementary Document**): one for hobby engagement (n = 43,346 from 15 countries) and another for hobby engagement frequency (n = 31,538 from 14 countries). Median follow-up ranged from 3.67 (IQR = 1.83, 8.67) years in Germany to 7.83 (IQR = 3.92, 11.33) years in the USA in the hobby engagement sample, and from 3.92 (IQR = 1.83, 8.67) years in Germany to 8.50 (IQR = 4.50, 11.50) years in the USA in the frequency sample. The mean age in the hobby engagement sample ranged from 71.88 years in China to 75.85 years in the USA; in the hobby engagement frequency sample, it ranged from 71.79 years in China to 74.99 years in the USA. Females represented the majority in most countries, except in China (50.65% male) and Germany (51.11% male) in the hobby engagement sample, and in China (63.98% male), Germany (50.94% male), Italy (52.60% male), and Spain (53.01% male) in the hobby engagement frequency sample. Across countries, the proportion of participants who were married or partnered ranged from 56.91% to 79.55% in the hobby-engagement sample and from 61.37% to 82.70% in the frequency sample. The corresponding country-level ranges for participants who were not working were 59.63–98.30% and 69.74–97.82%, respectively.

Baseline frailty differed across countries in both samples. The median FI ranged from 0.07 to 0.20 in the hobby engagement sample and from 0.07 to 0.21 in the frequency sample, while the prevalence of frailty ranged from 7.66% to 36.91% the former and from 7.06% to 40.62% in the latter sample, respectively (Table S10 in the **Online Supplementary Document**). Regarding the hobby engagement prevalence across countries (Figure S1 in the **Online Supplementary Document**), Sweden (96.71%) and Denmark (96.31%) had the highest engagement levels, followed by Switzerland (94.87%), Germany (92.57%), and Austria (92.09%), while China reported the lowest prevalence (20.63%).

### Association between baseline hobby engagement and subsequent FI

**Figure 2:**
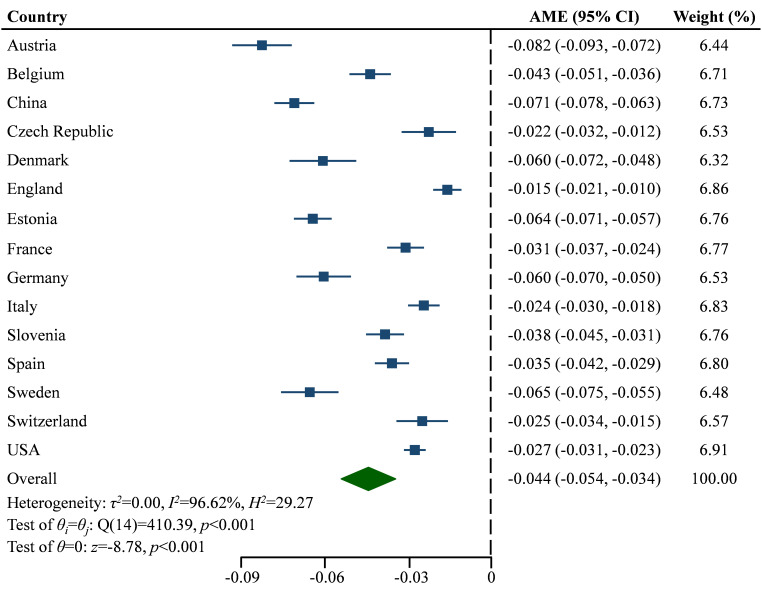
Association between hobby engagement and Frailty Index. AME – average marginal effect.

Despite high between-country heterogeneity (*I^2^* = 96.62%, *H^2^* = 29.27), hobby engagement was associated with a 0.044-point lower FI (95%CI = −0.054, −0.034; *P* < 0.001) after adjusting for covariates, equivalent to about 4.4% of the population mean (**Figure 2**). Country-specific estimates showed a consistent association between hobby engagement and FI, with varying magnitude across countries. Stronger associations were observed in Austria, China, Sweden, and Estonia, while weaker ones were found in England, Czech Republic, Italy, and Switzerland, and the USA. Predicted standardised scores for FI showed a consistently protective associations, with lower FI trajectories among participants with hobby engagement (Figure S2 in the **Online Supplementary Document**). The correlation between hobby engagement prevalence and country-level factors showed positive associations with gross domestic product *per capita*, the World Happiness Index, digital skills score, and internet use rate, as well as a negative association with Gini index (Figure S3 in the **Online Supplementary Document**). Hobby engagement-FI association was not significantly related to any examined country-level factor (Figure S4 in the **Online Supplementary Document**).

### Subpopulation analyses

The association between hobby engagement and FI was broadly consistent across subpopulations, although AMEs showed slight variation (Figure S5 in the **Online Supplementary Document**). Specifically, stronger effects were identified among the oldest-old, females, participants with tertiary education, current non-smokers, current non-drinkers, physically inactive individuals, and participants with lower household wealth, with all associations being statistically significant at *P*-values <0.05. Differences by marital status, residence area, and co-residence with children were not statistically significant, possibly reflecting contextual heterogeneity or limited power in these strata (Table S11 in the **Online Supplementary Document**).

### Association between baseline hobby engagement frequency and FI

**Figure 3:**
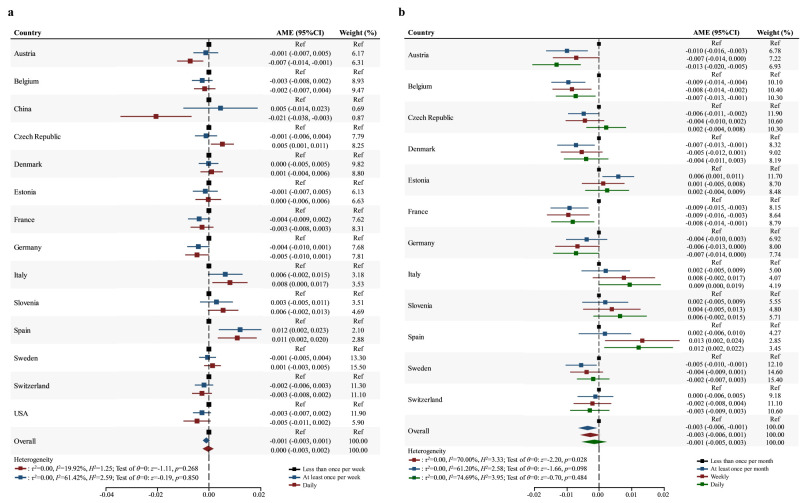
Association between hobby engagement frequency and Frailty Index across countries. **Panel A.** Use of three engagement frequency categories: less than once per week, at least once per week, and daily. **Panel B.** Use of four engagement frequency categories: less than once per month, at least once per month, weekly, and daily. The graph displays AME with 95% CI and corresponding weights. The reference category for comparison is ‘less than once per week’. AME – average marginal effect, CI – confidence interval.

Compared with hobby engagement less than once per week, neither weekly nor daily participation was associated with additional reductions in FI (**Figure 3**, Panel A). In a further analysis using four frequency categories, engagement at least monthly was associated with a significantly lower FI compared with engagement less than once per month (pooled AME = −0.003; 95% CI = −0.006, −0.001; *P* = 0.028). However, weekly or daily participation conferred no additional benefits (**Figure 3**, Panel B).

### Sensitivity analyses

Sensitivity analyses largely confirmed the main findings (Figures S6–22 in the **Online Supplementary Document**). Hobby engagement was consistently associated with lower subsequent FI, with pooled AMEs ranging from −0.056 to −0.036 (*P* < 0.05) and a lower risk of incident frailty in the Cox model (HR = 0.84; 95% CI = 0.77, 0.91; *P* < 0.001). Regarding the association between hobby engagement frequency and FI, sensitivity analyses confirmed that compared a frequency of less than once per month, only engagement at least monthly was associated with a significantly lower FI, except that weekly participation yielded an additional incremental AME in lagged models and was associated with a significantly lower risk of incident frailty in the Cox regression model (HR = 0.82; 95% CI = 0.73, 0.92; *P* < 0.001). Across the different sensitivity analyses, the pooled AMEs for engagement at least once per month ranged from −0.005 to −0.003 (all *P* < 0.05)

## DISCUSSION

In this cross-national analysis of adults aged ≥65 years across 15 countries, hobby engagement was consistently associated with subsequent lower FI. The strength of this association varied across countries and subpopulations. Regarding hobby engagement frequency, moderate engagement may be sufficient to confer benefit. A set of sensitivity analyses confirmed the robustness of our findings. To our knowledge, this is the first study to integrate individual-level longitudinal data from multiple cohorts across continents to examine the association between hobby engagement and frailty.

Our findings of lower subsequent frailty among older adults who engaged in hobbies are broadly consistent with previous longitudinal studies linking leisure or hobby participation to lower frailty [9,30,31]. Several psychosocial mechanisms may help explain this relationship. Psychosocially, hobbies may strengthen social connectedness and belonging, thereby reducing loneliness and social isolation [32], both of which are established risk factors for frailty. Hobby engagement may also buffer the harmful effects of chronic stress by alleviating stress, promoting positive affect, and enhancing self-efficacy [33]. Cognitively, hobbies provide sustained cognitive stimulation and opportunities for skill development, which may help preserve cognitive function [34]. Behaviourally, hobby engagement is often accompanied by healthier lifestyles, such as increased physical activity and greater routine regularity, indirectly strengthening resilience against frailty [35]. In addition, hobbies may foster a stronger sense of purpose in life, a factor consistently linked to lower frailty risk [36]. Within the deficit accumulation model, these psychosocial, cognitive, behavioural, and biological benefits may work together to slow the accumulation of health deficits across multiple physiological and functional domains, thereby contributing to a lower FI over time.

Meta-regression analyses did not detect significant associations between country-level indicators and the AMEs of hobby engagement on FI. This indicates that cross-national variations in the hobby-FI association may not be explained by the country-level indicators examined in the current study, rather by unmeasured factors such as cultural norms, community structures, or policy contexts, warranting future research with more granular social and cultural measures.

The associations between hobby engagement and subsequent frailty varied across subpopulations. Consistent with prior findings [9], the stronger association among the oldest-old, physically inactive individuals, and those with lower household wealth suggests that hobbies may function as accessible compensatory resources, offering cognitive stimulation, social participation, and a sense of purpose to those with fewer alternative protective resources [37]. The stronger association among participants with higher education may reflect greater cognitive reserve and higher health and digital literacy that enable more effective use of health-promoting resources embedded in hobbies [38]. In addition, the association was more pronounced among current non-smokers and non-drinkers, possibly because the benefits of hobby engagement are less likely to be offset by harmful health behaviours and may be reinforced by healthier lifestyle profiles. No significant sex difference was observed, suggesting similar pathways across men and women. However, these subpopulation differences should be interpreted cautiously. Their biological plausibility remains unclear, and some may reflect residual confounding or differential participation patterns rather than true effect modification. Further studies are needed to clarify these subpopulation patterns.

Notably, hobby engagement frequency did not show a simple dose-response relationship: compared with less frequent participation, monthly engagement was associated with lower FI, while more frequent engagement showed no clear additional benefit. One possible explanation is that once a basic threshold is reached, the psychological, social, and cognitive stimulation derived from hobbies may already fulfil essential needs for maintaining resilience, leaving limited scope for further gains [39]. More frequent engagement might also displace other health-promoting activities, such as exercise or outdoor pursuits, or even contribute to fatigue, thereby weakening incremental benefits [40]. However, this pattern may also reflect imprecise measurement of engagement frequency or selective participation, as weekly or daily participants may differ in underlying health or social circumstances [41]. These results imply that maintaining hobby engagement at an appropriate frequency may be an effective approach for frailty prevention, although further research is needed to clarify this finding and the underlying mechanisms.

Our findings have several policy and public health implications. First, hobby engagement may represent a low-cost, scalable, and culturally adaptable strategy for active-ageing frameworks, especially in rapidly ageing settings with limited healthcare resources. However, although the association was statistically significant, its practical significance remains unclear because we did not examine whether the FI difference translated into clinically meaningful reductions in adverse outcomes. Second, greater attention could be directed to vulnerable groups, including socially isolated, physically inactive, and socioeconomically disadvantaged older adults. Finally, policy efforts should focus on reducing barriers to participation and supporting regular engagement rather than simply encouraging higher frequency.

There are several limitations in this study. First, we cannot establish causal inference between hobby engagement and frailty because of the observational design of our study. Research with stronger causal designs, including quasi-experimental approaches and intervention studies, are needed to clarify whether promoting hobby engagement can directly reduce frailty risk. Second, although hobby engagement was harmonized across cohorts, the original survey items were not fully comparable. The CHARLS and SHARE provided examples of hobbies, while the ELSA and HRS did not, which may have led to differences in interpretation and affected the consistency of responses. Although sensitivity analyses based on cohort-specific measurement approaches yielded consistent results, misclassification due to incomplete measurement comparability cannot be entirely excluded. Third, hobby engagement was self-reported, introducing the possibility of recall or information bias. Fourth, despite adjustment for multiple confounders, residual confounding cannot be excluded due to data availability. Fifth, although the observed association was statistically significant, the clinical relevance of the effect size remains uncertain because we did not directly assess whether the observed difference in FI corresponds to meaningful reductions in disability, hospitalisation, mortality, or other adverse outcomes. Future studies incorporating specific clinical outcomes are needed to further clarify its clinical significance. Finally, in ELSA, mortality data were available up to wave 6, while frailty was followed until wave 10, which may have led to misclassification of follow-up time.

## CONCLUSIONS

In this cross-national study, hobby engagement was consistently associated with lower FI among older adults. The association appeared to plateau beyond a modest level of engagement. These findings suggest that hobby engagement at an appropriate frequency may represent a simple and scalable correlate of lower frailty in later life and could be considered in public health and social care policies for older adults.

## Additional material


Online Supplementary Document

## Data Availability

**Data availability:** The data sets used in this study can be found at the following links: CHARLS: https://charls.charlsdata.com/pages/data/111/en.html; ELSA: https://beta.ukdataservice.ac.uk/datacatalogue/series/series?id=200011; HRS: https://hrsdata.isr.umich.edu/data-products/rand; SHARE: https://www.share-project.org/data-access.html.
