# Supplementary material for: Association between hobby engagement and frailty among adults aged 65 years and older in 15 countries: findings from four prospective cohort studies
Source: J Glob Health. 2026 Jul 24;16:04208. doi: 10.7189/jogh.16.04208 (PMC13397102; doi:10.7189/jogh.16.04208)
Supplement: Online Supplementary Document [file jogh-16-04208-s001.pdf]

Supplement to: Fu T, Zhao Y, Luo Y, Wang H, Su L, Miao Y, Li J, Qin Y, Guo R. Association between hobby engagement and frailty among adults aged 65 years and older in 15 countries: findings from four prospective cohort studies. J Glob Health. 2026;16:04208.

CONTENTS

Additional Methods **3**
Additional Method 1. Meta-regressions with seven country-level factors 3
Additional Table 1. Adherence to JoGH's GRABDROP guidelines item 4
Additional Results of the Selected Studies **6**
Additional Table 2. Regions, countries, and waves included in analyses from CHARLS, ELSA, HRS, and SHARE 6
Additional Table 3. The items used to construct the Frailty Index in analyses from CHARLS, ELSA, HRS, and SHARE 7
Additional Table 4. Harmonization of hobby engagement and hobby engagement frequency in the CHARLS, ELSA, HRS, and SHARE 8
Additional Table 5. Harmonization of covariates in the CHARLS, ELSA, HRS, and SHARE 9
Additional Results from the Analysis using the Multiple Imputation Datasets ... **10**
Additional Table 6. The proportion of missing data for variables in the hobby engagement analytical sample 10
Additional Table 7. The proportion of missing data for variables in the hobby engagement frequency analytical sample 11
Additional Table 8. Baseline characteristics of the population for hobby engagement and Frailty Index by countries 12
Additional Table 9. Baseline characteristics of the population for hobby engagement frequency and Frailty Index by countries 14
Additional Table 10. Frailty distribution at baseline in both the hobby engagement and the hobby engagement frequency analytical samples 15
Additional Table 11. Associations of hobby engagement with Frailty Index by age, sex, marital status, education, residence area, smoking history, alcohol drinking history, weekly moderate to vigorous physical activity, labor force status, co-residence with children, and household wealth 17
Additional Fig.1 Levels of hobby engagement prevalence among adults aged 65 and above across 15 countries 21
Additional Fig.2 Predicted standardized scores for Frailty Index over time by baseline hobby engagement 23
Additional Fig.3 Pearson correlations between hobby engagement prevalence and country-level factors 24
Additional Fig.4 Bubble plot with fitted meta regression line of the average marginal effect for Frailty Index and country-level factors 25
Additional Results of Sensitivity Analysis **27**

Additional Fig.6 Flowchart of sample selection for analysis using complete cases ...27
Additional Fig.7 Association between hobby engagement and Frailty Index using complete cases ...28
Additional Fig.8 Association between hobby engagement frequency and Frailty Index using complete cases ...29
Additional Fig.9 Flowchart of sample selection for analysis among adults aged 60 and above ...30
Additional Fig.10 Association between hobby engagement and Frailty Index among adults aged 60 and above ...31
Additional Fig.11 Association between hobby engagement frequency and Frailty Index among adults aged 60 and above ...32
Additional Fig.12 Association between hobby engagement and Frailty Index using the inverse probability weights of attrition ...33
Additional Fig.13 Association between hobby engagement frequency and Frailty Index using the inverse probability weights of attrition ...34
Additional Fig.14 Association between hobby engagement and Frailty Index using lagged models ...35
Additional Fig.15 Association between hobby engagement frequency and Frailty Index using lagged models ...36
Additional Fig.16 Association between hobby engagement and Frailty Index excluding data from ELSA and HRS ...37
Additional Fig.17 Association between hobby engagement frequency and Frailty Index excluding data from ELSA and HRS using three frequency categories: less than once per week, at least once per week, and daily ...38
Additional Fig.18 Association between hobby engagement and Frailty Index excluding data from CHARLS ...39
Additional Fig.19 Association between hobby engagement frequency and Frailty Index excluding data from CHARLS using three frequency categories: less than once per week, at least once per week, and daily ...40
Additional Fig.20 Flowchart of sample selection for analysis regarding incident frailty using the COX regression model ...41
Additional Fig.21 Association between hobby engagement and incident frailty ...42
Additional Fig.22 Association between hobby engagement frequency and incident frailty ...43
References ...44
Text S1. Explanation of authorship change statement ...45

Additional Methods

Additional Method 1. Meta-regressions with seven country-level factors

To explore potential explanations for cross-country differences, we collected six country-level indicators covering four dimensions: economic (gross domestic product [GDP] per capita in 2022 and the Gini index in 2018–2021), social and cultural (World Happiness Index in 2023, Internet use rate in 2021–2022, and digital skills among active population in 2019), and healthcare-related (healthy life expectancy [HALE] at birth in 2019). GDP per capita was used as an indicator of national economic performance and living standards, calculated as the total value of goods and services produced divided by the population. A higher GDP per capita typically indicates a wealthier country[1]. The Gini index quantifies income and wealth inequality on a 0–100 scale, with higher values reflecting greater disparity[2]. The World Happiness Index provided a measure of subjective well-being, where higher scores indicate greater life satisfaction[3]. The Internet use rate assesses the proportion of individuals using the internet, reflecting digital penetration and access[4]. Digital skills among active population were obtained from the Executive Opinion Survey, in which experts rated the adequacy of population-level competencies (e.g., computer literacy, basic coding, digital reading) on a scale of 0–100, with higher values denoting stronger digital capacity[5]. HALE at birth measures the average number of years a person can expect to live in good health by considering both life expectancy and years lived with disability or disease. A higher HALE reflects not only longer survival but also better population health, thereby serving as an indicator of healthcare system effectiveness and the broader health environment[6].

To explore the heterogeneity variance in meta-analysis, we conducted random-effect meta-regression analyses by entering each country-level indicator separately as the explanatory variable. Potential non-linear relationships between AMEs and these indicators were examined by fitting linear, quadratic, and cubic functional forms. The final specification was selected if the Q-test indicated statistical significance or if the proportion of explained variance improved by at least 10% relative to the linear model[7].

Additional Table 1. Adherence to JoGH's GRABDROP guidelines item.

**JoGH guideline items**
1. Please list all papers published by each co-author in previous 3 years that were based on secondary analysis of a big data repository
(1) Guo R, Zhang S, Fu T, Guan Y, Luo Y, Wu Y*. Association between body mass index and disability among older population in China: analysis of 2011-2020 data from the China Health and Retirement Longitudinal Study. J Glob Health. 2025;15:04277. Published 2025 Dec 5. doi:10.7189/jogh.15.04277
(2) Miao Y, Luo Y, Zhao Y, Wang H, Wu Y*. Temporal trends and machine learning prediction for chronic kidney disease due to hypertension in young adults from 1990 to 2021: an analysis of the global burden of disease study 2021. Ann Med. 2025;57(1):2537916. doi:10.1080/07853890.2025.2537916
2. Please explain the key elements of your study design and the use of the available datasets that make your study an original scientific contribution
The originality of this study lies in the harmonization of longitudinal data from four large population-based ageing cohorts (CHARLS, ELSA, HRS, and SHARE) across 15 countries, combined with the application of a two-stage individual participant data (IPD) meta-analysis. This cross-national analytic framework enables comparable analyses while preserving between-country heterogeneity, thereby providing more robust and generalizable evidence than analyses based on a single cohort.
Second, we used longitudinal follow-up data to assess the association between baseline hobby engagement and subsequent frailty, thereby improving temporal ordering compared with cross-sectional analyses.
Third, in addition to examining hobby engagement as a binary exposure, we further investigated engagement frequency and explored subgroup differences across demographic, socioeconomic, and behavioral characteristics. These analyses address important gaps in the existing literature, where hobby engagement has often been examined only in specific forms, within single-country settings, or without consideration of engagement frequency and population heterogeneity.
3. Please list all publications that addressed similar research questions in the same dataset and indicate where you cited them in your paper
The following publications addressed similar questions in the datasets used in this study:
(1) Li Y, Liu M, Li X, et al. Change of leisure activity participation and associations with cognitive frailty in older adults: A population-based longitudinal study. Arch Gerontol Geriatr. 2025;129:105651. doi:10.1016/j.archger.2024.105651
(2) Bone JK, Bu F, Fluharty ME, Paul E, Sonke JK, Fancourt D. Engagement in leisure activities and depression in older adults in the United States: Longitudinal evidence from the Health and Retirement Study. Soc Sci Med. 2022;294:114703. doi:10.1016/j.socscimed.2022.114703
(3) Weziak-Bialowolska D, Bialowolski P, Sacco PL. Mind-stimulating leisure activities: Prospective associations with health, wellbeing, and longevity. Front Public Health. 2023;11:1117822. Published 2023 Feb 17. doi:10.3389/fpubh.2023.1117822

(4) Kim ES, Whillans AV, Lee MT, Chen Y, VanderWeele TJ. Volunteering and Subsequent Health and Well-Being in Older Adults: An Outcome-Wide Longitudinal Approach. Am J Prev Med. 2020;59(2):176-186. doi:10.1016/j.amepre.2020.03.004

Among the aforementioned publications, the study by Li et al. Utilized the CHARLS data to explore the association between changes in leisure activity participation patterns and cognitive frailty among older adults. This literature has been cited as Reference No. 9 in the Introduction section of the revised manuscript.

The remaining three studies did not explore the relationship between hobby engagement and frailty, but examined related topics. Specifically, Bone et al. used longitudinal evidence from the HRS to investigate the association between engagement in leisure activities and depression among older adults in the United States. Weziak-Bialowolska et al. employed the SHARE data to examine the prospective associations between three mind-stimulating leisure activities (reading, doing number and word games, and playing cards and games) and 21 outcomes across physical health, wellbeing, daily life functioning, cognitive impairment, and longevity. By using the HRS data, Kim et al. found that volunteering could effectively increase physical activity levels, improve psychosocial outcomes, and reduce the risks of disability and mortality in older adults. These three studies have been cited as Reference Nos. 33, 36, and 39, respectively, in the Discussion section of the revised manuscript.

It should also be noted that while the Introduction section incorporates a review of prior literature and cites analogous studies based on distinct databases (e.g., CLHLS, JAGES), detailed discussions of these studies are omitted from the present paper.

In summary, to our knowledge, no previous study has simultaneously integrated data from CHARLS, ELSA, HRS, and SHARE to conduct a harmonized cross-national longitudinal analysis of the association between hobby engagement and frailty, nor has any study thoroughly explored this relationship among subpopulations or the association between hobby frequency and frailty.

4. Please explain how you addressed multiple testing through an appropriately rigorous statistical threshold and indicate this in the methods section

We addressed multiple testing by applying the Benjamini-Hochberg false discovery rate procedure to the interaction *p-values* in subpopulation analyses. This method provides a more rigorous statistical threshold for multiple comparisons than reliance on unadjusted *p-values* alone and helps reduce the likelihood of false-positive findings. The corresponding statement was presented in the Methods section.

We also interpreted subpopulation findings cautiously as exploratory and expanded explanation to avoid overinterpretation of isolated statistically significant results.

5. Please declare to what extent have AI chatbots been used in developing your paper and to which parts of the paper did they contribute

No AI chatbots were used in developing this manuscript.

Additional Results of the Selected Studies

Additional Table 2. Regions, countries, and waves included in analyses from CHARLS, ELSA, HRS, and SHARE.

Study	Regions	Countries	Waves
CHARLS	Eastern Asia	China	Wave 1-4 (2011-2019)
ELSA	Western Europe	England	Wave 5-10 (2010-2021)
HRS	Northern America	United States	Wave 10-16 (2010-2023)
SHARE	Northern Europe	Denmark	Wave 4-9 (2011-2022)
		Sweden	
	Western Europe	Austria	
		Belgium	
		France	
		Germany	
		Switzerland	
	Southern Europe	Italy	
		Slovenia	
		Spain	
	Eastern Europe	Estonia	
	Central Europe	Czech Republic	

CHARLS, China Health and Retirement Longitudinal Study; ELSA, English Longitudinal Study of Ageing; HRS, Health and Retirement Study; SHARE, Survey of Health, Ageing and Retirement in Europe.

Additional Table 3. The items used to construct the Frailty Index in analyses from CHARLS, ELSA, HRS, and SHARE.

No.	Description of the items				Cut-off values
	CHARLS	ELSA	HRS	SHARE	
1	Self-reported hypertension diagnosed by physicians				Yes=1, No=0
2	Self-reported diabetes diagnosed by physicians				Yes=1, No=0
3	Self-reported cancer diagnosed by physicians				Yes=1, No=0
4	Self-reported arthritis diagnosed by physicians				Yes=1, No=0
5	Self-reported heart diseases diagnosed by physicians				Yes=1, No=0
6	Self-reported stroke diagnosed by physicians				Yes=1, No=0
7	Self-reported chronic lung disease diagnosed by physicians				Yes=1, No=0
8	Self-reported any emotional, nervous, or psychiatric problems diagnosed by physicians				Yes=1, No=0
9	Self-reported memory-related disease, including Alzheimer's disease, dementia, and other serious memory impairment diagnosed by physicians				Yes=1, No=0
10	Self-reported eyesight (while using lenses if appropriate)				Poor or fair=1, excellent, very good, or good=0
11	Self-reported hearing (while using hearing aids if appropriate)				Poor or fair=1, excellent, very good, or good=0
12	Self-reported general health status				Poor or fair=1, excellent, very good, or good=0
13	Difficulty with dressing				Yes=1, No=0
14	Difficulty with bathing or showering				Yes=1, No=0
15	Difficulty with eating				Yes=1, No=0
16	Difficulty with getting in and out of bed				Yes=1, No=0
17	Difficulty with using the toilet				Yes=1, No=0
18	Difficulty with managing money				Yes=1, No=0
19	Difficulty with taking medication				Yes=1, No=0
20	Difficulty with shopping for groceries				Yes=1, No=0
21	Difficulty with preparing meals				Yes=1, No=0
22	Mobility: difficulty with walking 100 meters or one block				Yes=1, No=0
23	Mobility: difficulty with getting up from a chair after sitting for long periods				Yes=1, No=0
24	Mobility: difficulty with climbing several flights of stairs without resting				Yes=1, No=0
25	Mobility: difficulty with lifting or carrying weights over 10 pounds/jin				Yes=1, No=0
26	Mobility: difficulty with picking up a coin from the table				Yes=1, No=0
27	Mobility: difficulty with stooping, kneeling, or crouching				Yes=1, No=0
28	Mobility: difficulty with reaching arms above shoulder level				Yes=1, No=0
29	Depression: CESD-10 scale (0-30)	Depression: CESD-8 scale (0-8)		EURO-D scale (0-12)	CESD-10>10=1, ≤10=0 in the CHARLS; CESD-8≥4=1, <4=0 in the ELSA and HRS; EURO-D≥4=1,<4=0 in the SHARE
30	Cognition: immediate and delayed word recall + time orientation + serial 7's	Cognition: immediate and delayed word recall + time orientation + animal naming fluency test converted to 0-5 range in waves 5 & 7-10 (numeracy ability in wave 6)	Cognition: immediate and delayed word recall + time orientation + serial 7's		Participants who performed 1.5 standard deviations below the mean of the score in two or three tests were coded as 1, and other participants were coded as 0

CHARLS, China Health and Retirement Longitudinal Study; ELSA, English Longitudinal Study of Ageing; HRS, Health and Retirement Study; SHARE, Survey of Health, Ageing and Retirement in Europe; CESD, Center for Epidemiological Studies-Depression; EURO-D, European Depression scale.

Additional Table 4. Harmonization of hobby engagement and hobby engagement frequency in the CHARLS, ELSA, HRS, and SHARE.

Variable	Harmonized value	Measurements in four studies			
		CHARLS	ELSA	HRS	SHARE
**Hobby engagement**	Yes	Which of the social activities listed on this card - if any - have you done in the past month?<br>1. Played ma-jong, played chess, played cards or went to a community club.<br>2. Went to a sport, social or other club.<br>3. Took part in a community-related organization.<br>4. Did voluntary or charity work.<br>5. Attended an educational or training course.<br>• Any of the activities	Does the statement apply to you: I have a hobby or past-time?<br>• Yes	How often do you work on a hobby or project?<br>• Daily/several times a week/once a week/several times a month/at least once a month	Which of the activities listed on this card - if any - have you done in the past twelve months?<br>1. Done voluntary or charity work.<br>2. Attended an educational or training course.<br>3. Gone to a sport, social or other kind of club.<br>4. Taken part in a political or community-related organization.<br>5. Read books, magazines or newspapers.<br>6. Did word or number games such as crossword puzzles or Sudoku.<br>7. Played cards or games such as chess.<br>• Any of the activities
	No	• None of the activities	• No	• Not in the last month/never	• None of the activities
**Hobby engagement frequency**	Daily	How often in the last month did you do voluntary or charity work/play ma-jong, chess, cards or go to a community club/ go to a sport, social or other kind of club/take part in a community-related organization/attended an educational or training course?<br>• Almost daily	Not available	How often do you work on a hobby or project?<br>• Daily	How often in the past twelve months did you do voluntary or charity work/attend an educational or training course/go to a sport, social or other kind of club/take part in a political or community-related organization/read books, magazines or newspapers/do word or number games such as crossword puzzles or Sudoku/ play cards or games such as chess?<br>• Almost daily
	At least once per week	• Almost every week		• Several times a week<br>• Once a week	• Almost every week
	Less than once per week	• Not regularly		• Several times a month<br>• At least once a month	• Almost every month<br>• Less often

CHARLS, China Health and Retirement Longitudinal Study; ELSA, English Longitudinal Study of Ageing; HRS, Health and Retirement Study; SHARE, Survey of Health, Ageing and Retirement in Europe.

Additional Table 5. Harmonization of covariates in the CHARLS, ELSA, HRS, and SHARE.

Variable	Harmonized value	Measurements in four studies			
		CHARLS	ELSA	HRS	SHARE
**Age, years old**	Continuous	The respondent's age			
**Sex**	Male<br>Female	The respondent's sex			
**Marital status**	Married or partnered	Married, separated, or partnered			
	Unmarried and others	Divorced, widowed, and never married			
**Education**	Primary	Less than upper secondary education			
	Secondary	Upper secondary education & vocational training			
	Tertiary	Tertiary education			
**Smoking history**	Never smoker	The respondent has never smoked.			
	Former smoker	The respondent used to smoke but has quit now.			
	Current smoker	The respondent currently smokes.			
**Alcohol drinking history**	Never drinker	The respondent has never drunk.			
	Former drinker	The respondent used to drink but has quit now.			
	Current drinker	The respondent currently drinks.			
**Weekly moderate to vigorous physical activity**	Yes	Number of days of vigorous or moderate physical activity for at least 10 minutes.<br>• 1-7 day	Frequency of taking part in vigorous or moderate physical activity.<br>• One to three times a month<br>• Once a week<br>• More than once a week	Frequency of taking part in vigorous or moderate physical activity.<br>• One to three times a month<br>• Once a week<br>• More than once a week<br>• Every day	Frequency of taking part in vigorous or moderate physical activity.<br>• One to three times a month<br>• Once a week<br>• More than once a week
	No	• 0 day	• Hardly ever or never		
**Employment status**	Working	Employed or self-employed in agricultural or nonagricultural sectors or nonagricultural unpaid family business	Employed or self-employed	Working full-time or part-time	Employed or self-employed
	Not working	Retired, never worked, or unemployed	Partly retired, retired, disabled, unemployed, or looking after home or family	Partly retired, retired disabled, not in the labor force, or unemployed	Retired, unemployed, permanently sick or disabled, or homemaker
**Co-residence with children**	Yes<br>No	Whether the respondent lives with his/her children currently.			
**Residence area**	Urban<br>Rural	Urban vs. rural based on household address.			
**Household wealth**	Low level: tertile 1<br>Middle level: tertile 2<br>High level: tertile 3	The total annual income of all household members, including wages, salaries, benefits, and any other income sources, divided by the number of household members, expressed in local currencies.			

CHARLS, China Health and Retirement Longitudinal Study; ELSA, English Longitudinal Study of Ageing; HRS, Health and Retirement Study; SHARE, Survey of Health, Ageing and Retirement in Europe.

Additional Results from the Analysis using the Multiple Imputation Datasets

Additional Table 6. The proportion of missing data for variables in the hobby engagement analytical sample.

Countries	Marital status	Education	Smoking history	Alcohol drinking history	Weekly moderate to vigorous physical activity	Labor force status	Co-residence with children	Residence status	Household wealth
Austria (N=2339)	351 (15.01%)	387 (16.55%)	540 (23.09%)	720 (30.78%)	721 (30.83%)	72 (3.08%)	767 (32.79%)	431 (18.43%)	720 (30.78%)
Belgium (N=2757)	469 (17.01%)	503 (18.24%)	848 (30.76%)	874 (31.70%)	874 (31.70%)	173 (6.27%)	942 (34.17%)	400 (14.51%)	873 (31.66%)
China (N=1682)	1 (0.06%)	0 (0.00%)	338 (20.1%)	5 (0.3%)	531 (31.57%)	11 (0.65%)	10 (0.59%)	0 (0.00%)	552 (32.82%)
Czech Republic (N=3280)	149 (4.54%)	173 (5.27%)	842 (25.67%)	1036 (31.59%)	1031 (31.43%)	92 (2.80%)	1078 (32.87%)	733 (22.35%)	1031 (31.43%)
Denmark (N=1733)	99 (5.71%)	113 (6.52%)	574 (33.12%)	517 (29.83%)	516 (29.77%)	71 (4.10%)	548 (31.62%)	194 (11.19%)	516 (29.77%)
England (N=1839)	1 (0.05%)	7 (0.38%)	261 (14.19%)	549 (29.85%)	0 (0.00%)	2 (0.11%)	111 (6.04%)	0 (0.00%)	68 (3.70%)
Estonia (N=3162)	328 (10.37%)	353 (11.16%)	759 (24.00%)	993 (31.40%)	989 (31.28%)	93 (2.94%)	942 (29.79%)	670 (21.19%)	989 (31.28%)
France (N=2691)	65 (2.42%)	79 (2.94%)	577 (21.44%)	651 (24.19%)	649 (24.12%)	85 (3.16%)	718 (26.68%)	323 (12.00%)	648 (24.08%)
Germany (N=2747)	65 (2.37%)	75 (2.73%)	706 (25.70%)	939 (34.18%)	939 (34.18%)	49 (1.78%)	770 (28.03%)	418 (15.22%)	939 (34.18%)
Italy (N=2874)	500 (17.40%)	546 (19.00%)	769 (26.76%)	899 (31.28%)	896 (31.18%)	19 (0.66%)	958 (33.33%)	749 (26.06%)	895 (31.14%)
Slovenia (N=2356)	255 (10.82%)	281 (11.93%)	496 (21.05%)	749 (31.79%)	749 (31.79%)	77 (3.27%)	763 (32.39%)	538 (22.84%)	749 (31.79%)
Spain (N=3169)	222 (7.01%)	238 (7.51%)	733 (23.13%)	997 (31.46%)	995 (31.40%)	69 (2.18%)	993 (31.33%)	657 (20.73%)	994 (31.37%)
Sweden (N=2827)	147 (5.20%)	169 (5.98%)	793 (28.05%)	958 (33.89%)	957 (33.85%)	108 (3.82%)	972 (34.38%)	537 (19.00%)	955 (33.78%)
Switzerland (N=1540)	44 (2.86%)	48 (3.12%)	502 (32.60%)	461 (29.94%)	460 (29.87%)	69 (4.48%)	419 (27.21%)	268 (17.40%)	460 (29.87%)
USA (N=8350)	49 (0.59%)	127 (1.52%)	123 (1.47%)	36 (0.43%)	16 (0.19%)	48 (0.57%)	3 (0.04%)	0 (0.00%)	2514 (30.11%)

Additional Table 7. The proportion of missing data for variables in the hobby engagement frequency analytical sample.

Countries	Marital status	Education	Smoking history	Alcohol drinking history	Weekly moderate to vigorous physical activity	Labor force status	Co-residence with children	Residence status	Household wealth
Austria (N=2152)	331 (15.38%)	364 (16.91%)	515 (23.93%)	664 (30.86%)	665 (30.90%)	63 (2.93%)	575 (26.72%)	400 (18.59%)	631 (29.93%)
Belgium (N=2465)	440 (17.85%)	471 (19.11%)	571 (23.16%)	610 (24.65%)	783 (31.76%)	144 (5.84%)	670 (27.18%)	368 (14.93%)	709 (28.76%)
China (N=347)	0 (0.00%)	0 (0.00%)	96 (27.67%)	2 (0.58%)	108 (31.12%)	0 (0.00%)	2 (0.58%)	0 (0.00%)	116 (33.43%)
Czech Republic (N=2928)	143 (4.88%)	166 (5.67%)	777 (26.54%)	914 (31.22%)	850 (29.03%)	77 (2.63%)	792 (27.05%)	673 (22.98%)	750 (25.61%)
Denmark (N=1668)	96 (5.76%)	110 (6.59%)	452 (27.10%)	470 (28.18%)	509 (30.52%)	64 (3.84%)	538 (32.25%)	191 (11.45%)	509 (30.52%)
Estonia (N=2798)	310 (11.08%)	332 (11.87%)	701 (25.05%)	858 (30.66%)	855 (30.56%)	76 (2.72%)	896 (32.02%)	615 (21.98%)	955 (34.13%)
France (N=2247)	55 (2.45%)	66 (2.94%)	505 (22.47%)	565 (25.14%)	564 (25.10%)	66 (2.94%)	617 (27.46%)	277 (12.33%)	563 (25.06%)
Germany (N=2542)	64 (2.52%)	74 (2.91%)	664 (26.12%)	793 (31.20%)	793 (31.20%)	39 (1.53%)	816 (32.10%)	390 (15.34%)	763 (30.02%)
Italy (N=1498)	308 (20.56%)	335 (22.36%)	463 (30.91%)	480 (32.04%)	478 (31.91%)	9 (0.60%)	473 (31.58%)	380 (25.37%)	478 (31.91%)
Slovenia (N=1791)	216 (12.06%)	239 (13.34%)	407 (22.72%)	554 (30.93%)	554 (30.93%)	57 (3.18%)	563 (31.43%)	418 (23.34%)	554 (30.93%)
Spain (N=1613)	123 (7.63%)	132 (8.18%)	433 (26.84%)	428 (26.53%)	427 (26.47%)	24 (1.49%)	483 (29.94%)	301 (18.66%)	427 (26.47%)
Sweden (N=2734)	142 (5.19%)	162 (5.93%)	769 (28.13%)	737 (26.96%)	736 (26.92%)	99 (3.62%)	648 (23.70%)	522 (19.09%)	734 (26.85%)
Switzerland (N=1459)	43 (2.95%)	47 (3.22%)	419 (28.72%)	341 (23.37%)	340 (23.30%)	63 (4.32%)	395 (27.07%)	258 (17.68%)	340 (23.30%)
USA (N=5296)	33 (0.62%)	89 (1.68%)	84 (1.59%)	24 (0.45%)	11 (0.21%)	35 (0.66%)	3 (0.06%)	0 (0.00%)	2085 (39.37%)

Additional Table 8. Baseline characteristics of the population for hobby engagement and Frailty Index by countries.

Characteristics	Austria (N=2339)	Belgium (N=2757)	China (N=1682)	Czech Republic (N=3280)	Denmark (N=1733)	England (N=1839)	Estonia (N=3162)	France (N=2691)
Age	73.60 ± 6.35	74.35 ± 6.97	71.88 ± 5.61	72.80 ± 6.30	73.39 ± 6.91	74.19 ± 6.50	73.88 ± 6.09	74.72 ± 7.01
Male	998 (42.67%)	1259 (45.67%)	852 (50.65%)	1407 (42.90%)	837 (48.30%)	681 (37.03%)	1264 (39.97%)	1133 (42.10%)
Marital status								
Married or partnered	1473 (62.98%)	1887 (68.44%)	1226 (72.89%)	2050 (62.50%)	1230 (70.98%)	1069 (58.13%)	1973 (62.40%)	1708 (63.47%)
Unmarried and others	866 (37.02%)	870 (31.56%)	456 (27.11%)	1230 (37.50%)	503 (29.02%)	770 (41.87%)	1189 (37.60%)	983 (36.53%)
Education								
Primary	735 (31.42%)	1382 (50.13%)	1568 (93.22%)	1343 (40.95%)	453 (26.14%)	944 (51.33%)	1310 (41.43%)	1592 (59.16%)
Secondary	1070 (45.75%)	629 (22.81%)	74 (4.40%)	1478 (45.06%)	705 (40.68%)	773 (42.03%)	1248 (39.47%)	674 (25.05%)
Tertiary	534 (22.83%)	746 (27.06%)	40 (2.38%)	459 (13.99%)	575 (33.18%)	122 (6.63%)	604 (19.10%)	425 (15.79%)
Smoking history								
Never smoker	1528 (65.33%)	1546 (56.08%)	969 (57.61%)	2050 (62.50%)	688 (39.70%)	723 (39.91%)	2049 (64.80%)	1654 (61.46%)
Former smoker	545 (23.30%)	929 (33.70%)	195 (11.59%)	765 (23.32%)	736 (42.47%)	920 (50.03%)	783 (24.76%)	839 (31.18%)
Current smoker	266 (11.37%)	282 (10.23%)	518 (30.80%)	465 (14.18%)	309 (17.83%)	196 (10.66%)	330 (10.44%)	198 (7.36%)
Alcohol drinking history								
Never drinker	1114 (47.63%)	1016 (36.85%)	983 (58.44%)	1887 (57.53%)	431 (24.87%)	543 (29.53%)	2538 (80.27%)	1144 (42.51%)
Former drinker	78 (3.33%)	42 (1.52%)	201 (11.95%)	243 (7.41%)	117 (6.75%)	869 (47.25%)	236 (7.46%)	36 (1.34%)
Current drinker	1147 (49.04%)	1699 (61.62%)	498 (29.61%)	1150 (35.06%)	1185 (68.38%)	427 (23.22%)	388 (12.27%)	1511 (56.15%)
Weekly moderate to vigorous physical activity	1025 (43.82%)	833 (30.21%)	366 (21.76%)	1005 (30.64%)	835 (48.18%)	1190 (64.71%)	1228 (38.84%)	729 (27.09%)
Labor force status								
Not working	2238 (95.68%)	2618 (94.96%)	1003 (59.63%)	2955 (90.09%)	1480 (85.40%)	1706 (92.77%)	2806 (88.74%)	2576 (95.73%)
Working	101 (4.32%)	139 (5.04%)	679 (40.37%)	325 (9.91%)	253 (14.60%)	133 (7.23%)	356 (11.26%)	115 (4.27%)
Co-residence with children	258 (11.03%)	215 (7.80%)	705 (41.91%)	398 (12.13%)	43 (2.48%)	228 (12.40%)	473 (14.96%)	231 (8.58%)
Residence status								
Urban	1250 (55.04%)	2002 (74.04%)	641 (38.11%)	2261 (72.42%)	1343 (78.22%)	-	2175 (71.12%)	1449 (55.35%)
Rural	1021 (44.96%)	702 (25.96%)	1041 (61.89%)	861 (27.58%)	374 (21.78%)	-	883 (28.88%)	1169 (44.65%)
Household wealth								
Low level	558 (23.86%)	332 (12.04%)	658 (39.12%)	1463 (44.60%)	182 (10.50%)	869 (47.25%)	1658 (52.44%)	405 (15.05%)
Middle level	1312 (56.09%)	1188 (43.09%)	747 (44.41%)	1529 (46.62%)	621 (35.83%)	668 (36.32%)	1067 (33.74%)	1221 (45.37%)
High level	469 (20.05%)	1237 (44.87%)	277 (16.47%)	288 (8.78%)	930 (53.66%)	302 (16.42%)	437 (13.82%)	1065 (39.58%)
Follow-up periods (years)	5.92 (1.83, 10.42)	5.83 (1.83, 8.92)	6.92 (4.00, 7.00)	5.75 (1.83, 8.67)	6.25 (2.00, 8.67)	6.42 (3.63, 9.13)	6.58 (2.67, 10.83)	3.92 (1.82, 8.67)

Additional Table 8. (continued).

Characteristics	Germany (N=2747)	Italy (N=2874)	Slovenia (N=2356)	Spain (N=3169)	Sweden (N=2827)	Switzerland (N=1540)	USA (N=8350)
Age	73.54 ± 6.24	73.53 ± 6.42	74.01 ± 6.55	75.24 ± 7.11	73.43 ± 6.72	73.32 ± 6.54	75.85 ± 7.13
Male	1404 (51.11%)	1396 (48.57%)	1073 (45.54%)	1491 (47.05%)	1355 (47.93%)	742 (48.18%)	3469 (41.54%)
Marital status							
Married or partnered	2123 (77.28%)	2249 (78.25%)	1701 (72.20%)	2521 (79.55%)	2162 (76.48%)	1166 (75.71%)	4752 (56.91%)
Unmarried and others	624 (22.72%)	625 (21.75%)	655 (27.80%)	648 (20.45%)	665 (23.52%)	374 (24.29%)	3598 (43.09%)
Education							
Primary	452 (16.45%)	2320 (80.72%)	965 (40.96%)	2838 (89.56%)	1365 (48.28%)	426 (27.66%)	1771 (21.21%)
Secondary	1493 (54.35%)	396 (13.78%)	1050 (44.57%)	134 (4.23%)	758 (26.81%)	910 (59.09%)	4945 (59.22%)
Tertiary	802 (29.02%)	158 (5.50%)	341 (14.47%)	197 (6.22%)	704 (24.90%)	204 (13.25%)	1634 (19.57%)
Smoking history							
Never smoker	1564 (56.93%)	1778 (61.86%)	1622 (68.85%)	2192 (69.17%)	1353 (47.86%)	875 (56.82%)	3583 (42.91%)
Former smoker	873 (31.78%)	785 (27.31%)	585 (24.83%)	765 (24.14%)	1195 (42.27%)	449 (29.16%)	4018 (48.12%)
Current smoker	310 (11.29%)	311 (10.82%)	149 (6.32%)	212 (6.69%)	279 (9.87%)	216 (14.03%)	749 (8.97%)
Alcohol drinking history							
Never drinker	1215 (44.23%)	1610 (56.02%)	1388 (58.91%)	2166 (68.35%)	1292 (45.70%)	494 (32.08%)	4399 (52.68%)
Former drinker	114 (4.15%)	58 (2.02%)	41 (1.74%)	42 (1.33%)	45 (1.59%)	48 (3.12%)	573 (6.86%)
Current drinker	1418 (51.62%)	1206 (41.96%)	927 (39.35%)	961 (30.33%)	1490 (52.71%)	998 (64.81%)	3378 (40.46%)
Weekly moderate to vigorous physical activity	1301 (47.36%)	786 (27.35%)	1007 (42.74%)	664 (20.95%)	1430 (50.58%)	740 (48.05%)	5099 (61.07%)
Labor force status							
Not working	2467 (89.81%)	2751 (95.72%)	2316 (98.30%)	3055 (96.40%)	2246 (79.45%)	1311 (85.13%)	7011 (83.96%)
Working	280 (10.19%)	123 (4.28%)	40 (1.70%)	114 (3.60%)	581 (20.55%)	229 (14.87%)	1339 (16.04%)
Co-residence with children	223 (8.12%)	622 (21.64%)	533 (22.62%)	687 (21.68%)	65 (2.30%)	105 (6.82%)	1970 (23.59%)
Residence status							
Urban	1613 (59.90%)	1743(63.50%)	1018 (45.30%)	2816 (92.75%)	2200 (82.27%)	644 (43.31%)	5539 (67.42%)
Rural	1080 (40.10%)	1002 (36.50%)	1229 (54.70%)	220 (7.25%)	474 (17.73%)	843 (56.69%)	2677 (32.58%)
Household wealth							
Low level	429 (15.62%)	791 (27.52%)	1003 (42.57%)	1218 (38.43%)	207 (7.32%)	94 (6.10%)	1744 (20.89%)
Middle level	1459 (53.11%)	1411 (49.10%)	1068 (45.33%)	1303 (41.12%)	958 (33.89%)	484 (31.43%)	3087 (36.97%)
High level	859 (31.27%)	672 (23.38%)	285 (12.10%)	648 (20.45%)	1662 (58.79%)	962 (62.47%)	3519 (42.14%)
Follow-up periods (years)	3.67 (1.83, 8.67)	6.08 (1.83, 9.08)	6.33 (1.92, 8.67)	4.25 (2.08, 7.00)	5.92 (1.67, 8.58)	6.00 (2.00, 10.50)	7.83 (3.92, 11.33)

CHARLS, China Health and Retirement Longitudinal Study; ELSA, English Longitudinal Study of Ageing; HRS, Health and Retirement Study; SHARE, Survey of Health, Ageing and Retirement in Europe.

Additional Table 9. Baseline characteristics of the population for hobby engagement frequency and Frailty Index by countries.

Characteristics	Austria (N=2152)	Belgium (N=2465)	China (N=347)	Czech Republic (N=2928)	Denmark (N=1668)	Estonia (N=2798)	France (N=2247)
Age	73.42 ± 6.25	74.03 ± 6.79	71.79 ± 5.38	72.50 ± 6.09	73.24 ± 6.81	73.48 ± 5.95	74.40 ± 6.89
Male	936 (43.49%)	1127 (45.72%)	222 (63.98%)	1241 (42.38%)	797 (47.78%)	1121 (40.06%)	933 (41.52%)
Marital status							
Married or partnered	1377 (63.99%)	1712 (69.45%)	258 (74.35%)	1847 (63.08%)	1189 (71.28%)	1769 (63.22%)	1457 (64.84%)
Unmarried and others	775 (36.01%)	753 (30.55%)	89 (25.65%)	1081 (36.92%)	479 (28.72%)	1029 (36.78%)	790 (35.16%)
Education							
Primary	630 (29.28%)	1158 (46.98%)	294 (84.73%)	1136 (38.80%)	428 (25.66%)	1060 (37.88%)	1248 (55.54%)
Secondary	1005 (46.70%)	587 (23.81%)	28 (8.07%)	1352 (46.17%)	673 (40.35%)	1159 (41.42%)	598 (26.61%)
Tertiary	517 (24.02%)	720 (29.21%)	25 (7.20%)	440 (15.03%)	567 (33.99%)	579 (20.69%)	401 (17.85%)
Smoking history							
Never smoker	1387 (64.45%)	1355 (54.97%)	156 (44.96%)	1823 (62.26%)	664 (39.81%)	1802 (64.40%)	1364 (60.70%)
Former smoker	515 (23.93%)	858 (34.81%)	52 (14.99%)	686 (23.43%)	709 (42.51%)	701 (25.05%)	717 (31.91%)
Current smoker	250 (11.62%)	252 (10.22%)	139 (40.06%)	419 (14.31%)	295 (17.69%)	295 (10.54%)	166 (7.39%)
Alcohol drinking history							
Never drinker	996 (46.28%)	861 (34.93%)	169 (48.07%)	1644 (56.15%)	398 (23.86%)	2222 (79.41%)	901 (40.10%)
Former drinker	62 (2.88%)	37 (1.50%)	56 (16.14%)	227 (7.75%)	114 (6.83%)	216 (7.72%)	27 (1.20%)
Current drinker	1094 (50.84%)	1567 (63.57%)	122 (35.16%)	1057 (36.10%)	1156 (69.30%)	360 (12.87%)	1319 (58.70%)
Weekly moderate to vigorous physical activity	977 (45.40%)	777 (31.52%)	54 (15.56%)	951 (32.48%)	826 (49.52%)	1159 (41.42%)	666 (29.64%)
Labor force status							
Not working	2052 (95.35%)	2336 (94.77%)	242 (69.74%)	2621 (89.52%)	1420 (85.13%)	2455 (87.74%)	2143 (95.37%)
Working	100 (4.65%)	129 (5.23%)	105 (30.26%)	307(10.48%)	248 (14.87%)	343 (12.26%)	104 (4.63%)
Co-residence with children	230 (10.69%)	184 (7.46%)	124 (35.73%)	351 (11.99%)	38 (2.28%)	405 (14.47%)	172 (7.65%)
Residence status							
Urban	1186 (56.67%)	1807 (74.64%)	206 (59.37%)	2053 (73.61%)	1294 (78.33%)	1923 (71.25%)	1202 (54.91%)
Rural	907 (43.33%)	614 (25.36%)	141 (40.63%)	736 (26.39%)	358 (21.67%)	776 (28.75%)	987 (45.09%)
Household wealth							
Low level	464 (21.56%)	271 (10.99%)	89 (25.65%)	1244 (42.49%)	171 (10.25%)	1392 (49.75%)	268 (11.93%)
Middle level	1230 (57.16%)	1039 (42.15%)	166 (47.84%)	1417 (48.39%)	587 (35.19%)	987 (35.28%)	1021 (45.44%)
High level	458 (21.28%)	1155 (46.86%)	92 (26.51%)	267 (9.12%)	910 (54.56%)	419 (14.97%)	958 (42.63%)
Follow-up periods (years)	6.00 (1.83, 10.42)	5.92 (1.92, 9.08)	6.92 (4.00, 7.00)	5.83 (2.00, 8.75)	6.25 (2.00, 8.67)	6.83 (4.33, 10.92)	4.00 (1.67, 8.75)

Additional Table 9. (continued).

Characteristics	Germany (N=2542)	Italy (N=1498)	Slovenia (N=1791)	Spain (N=1613)	Sweden (N=2734)	Switzerland (N=1459)	USA (N=5296)
Age	73.31 ± 6.04	72.38 ± 5.77	73.65 ± 6.44	73.74 ± 6.45	73.32 ± 6.62	73.13 ± 6.44	74.99 ± 6.74
Male	1295 (50.94%)	788 (52.60%)	819 (45.73%)	855 (53.01%)	1297 (47.44%)	706 (48.39%)	2264 (42.75%)
Marital status							
Married or partnered	1984 (78.05%)	1197 (79.91%)	1329 (74.20%)	1334 (82.70%)	2096 (76.66%)	1110 (76.08%)	3250 (61.37%)
Unmarried and others	558 (21.95%)	301 (20.09%)	462 (25.80%)	279 (17.30%)	638 (23.34%)	349 (23.92%)	2046 (38.63%)
Education							
Primary	383 (15.07%)	1077 (71.90%)	629 (35.12%)	1347 (83.51%)	1307 (47.81%)	390 (26.73%)	760 (14.35%)
Secondary	1386 (54.52%)	293 (19.56%)	843 (47.07%)	103 (6.39%)	737 (26.96%)	870 (59.63%)	3264 (61.63%)
Tertiary	773 (30.41%)	128 (8.54%)	319 (17.81%)	163 (10.11%)	690 (25.24%)	199 (13.64%)	1272 (24.02%)
Smoking history							
Never smoker	1438 (56.57%)	819 (54.67%)	1199 (66.95%)	1056 (65.47%)	1309 (47.88%)	820 (56.20%)	2268 (42.82%)
Former smoker	826 (32.49%)	482 (32.18%)	472 (26.35%)	437 (27.09%)	1157 (42.32%)	438 (30.02%)	2571 (48.55%)
Current smoker	278 (10.94%)	197 (13.15%)	120 (6.70%)	120 (7.44%)	268 (9.80%)	201 (13.78%)	457 (8.63%)
Alcohol drinking history							
Never drinker	1081 (42.53%)	692 (46.19%)	1001 (55.89%)	949 (58.83%)	1229 (44.95%)	445 (30.50%)	2525 (47.68%)
Former drinker	108 (4.25%)	28 (1.87%)	29 (1.62%)	24 (1.49%)	43 (1.57%)	45 (3.08%)	399 (7.53%)
Current drinker	1353 (53.23%)	778 (51.94%)	761 (42.29%)	640 (39.68%)	1462 (53.47%)	969 (66.42%)	2372 (44.79%)
Weekly moderate to vigorous physical activity	1243 (48.90%)	503 (33.58%)	835 (46.62%)	414 (25.67%)	1409 (51.54%)	718 (49.21%)	3646 (68.84%)
Labor force status							
Not working	2271 (89.34%)	1411 (94.19%)	1752 (97.82%)	1535 (95.16%)	2165 (79.19%)	1238 (84.85%)	4307 (81.33%)
Working	271 (10.66%)	87 (5.81%)	39 (2.18%)	78 (4.84%)	569 (20.81%)	221 (15.15%)	989 (18.67%)
Co-residence with children	197 (7.75%)	272 (18.16%)	395 (22.05%)	331 (20.52%)	63 (2.30%)	95 (6.51%)	1052 (19.86%)
Residence status							
Urban	1504 (60.14%)	973 (66.92%)	800 (46.57%)	1435 (92.64%)	2133 (82.29%)	612 (43.44%)	3481 (66.58%)
Rural	997 (39.86%)	481 (33.08%)	918 (53.43%)	114 (7.36%)	459 (17.71%)	797 (56.56%)	1747 (33.42%)
Household wealth							
Low level	362 (14.24%)	281 (18.76%)	655 (36.57%)	457 (28.33%)	191 (6.99%)	80 (5.48%)	784 (14.80%)
Middle level	1345 (52.91%)	734 (49.00%)	890 (49.69%)	685 (42.27%)	918 (33.58%)	447 (30.64%)	1883 (35.56%)
High level	835 (32.85%)	483 (32.24%)	246 (13.74%)	471 (29.20%)	1625(59.44%)	932 (63.88%)	2629 (49.64%)
Follow-up periods (years)	3.92 (1.83, 8.67)	6.50 (2.00, 10.42)	6.50 (2.00, 8.67)	4.58 (2.17, 8.67)	5.92 (1.75, 8.58)	6.00 (2.00, 10.50)	8.50 (4.50, 11.50)

CHARLS, China Health and Retirement Longitudinal Study; ELSA, English Longitudinal Study of Ageing; HRS, Health and Retirement Study; SHARE, Survey of Health, Ageing and Retirement in Europe; PA, physical activity.

Additional Table 10. Frailty distribution at baseline in both the hobby engagement and the hobby engagement frequency analytical samples.

Countries	Hobby engagement sample				Hobby engagement frequency sample			
	**FI**	**Robust**	**Pre-frail**	**Frail**	**FI**	**Robust**	**Pre-frail**	**Frail**
Austria (N=2339)	0.10 (0.03, 0.21)	981 (41.94%)	902 (38.56%)	456 (19.50%)	0.10 (0.03, 0.20)	942 (43.77%)	839 (38.99%)	371 (17.24%)
Belgium (N=2757)	0.13 (0.07, 0.24)	906 (32.86%)	1220 (44.25%)	631 (22.89%)	0.13(0.07, 0.23)	848 (34.40%)	1130 (45.84%)	487 (19.76%)
China (N=1682)	0.20 (0.10, 0.30)	238 (21.28%)	750 (44.59%)	574 (34.13%)	0.13 (0.07, 0.23)	100 (28.82%)	177 (51.01%)	70 (20.17%)
Czech Republic (N=3280)	0.13 (0.07, 0.24)	1029 (31.37%)	1470 (44.82%)	781 (23.81%)	0.13 (0.07, 0.23)	970 (33.13%)	1363 (46.55%)	595 (20.32%)
Denmark (N=1733)	0.10 (0.03, 0.17)	857 (49.45%)	656 (37.85%)	220 (12.69%)	0.07 (0.03, 0.17)	846 (50.72%)	633 (37.95%)	189 (11.33%)
England (N=1839)	0.17 (0.07, 0.28)	537 (29.20%)	750 (40.78%)	552 (30.02%)	0.17 (0.10, 0.30)	582 (20.80%)	1322 (47.25%)	894 (31.95%)
Estonia (N=3162)	0.20 (0.10, 0.33)	605 (19.13%)	1419 (44.88%)	1138 (35.99%)	-	-	-	-
France (N=2691)	0.14 (0.07, 0.27)	818 (30.40%)	1197 (44.48%)	676 (25.12%)	0.13 (0.07, 0.24)	721 (32.09%)	1021 (45.44%)	505 (22.47%)
Germany (N=2747)	0.13 (0.07, 0.23)	1010 (36.77%)	1168 (42.52%)	569 (20.71%)	0.10 (0.07, 0.21)	971 (38.20%)	1104 (43.43%)	467 (18.37%)
Italy (N=2874)	0.17 (0.07, 0.30)	857 (29.82%)	1151 (40.05%)	866 (30.13%)	0.13 (0.07, 0.23)	530 (35.38%)	675 (45.06%)	293 (19.56%)
Slovenia (N=2356)	0.13 (0.07, 0.27)	748 (31.75%)	997 (42.32%)	611 (25.93%)	0.13 (0.07, 0.23)	620 (34.62%)	776 (43.33%)	395 (22.05%)
Spain (N=3169)	0.17 (0.07, 0.30)	927 (29.25%)	1233 (38.91%)	1006 (31.84%)	0.13 (0.07, 0.23)	604 (37.45%)	676 (41.91%)	333 (20.64%)
Sweden (N=2827)	0.10 (0.03, 0.17)	1352 (47.82%)	1136 (40.18%)	339 (11.99%)	0.10 (0.03, 0.17)	1337 (48.90%)	1101 (40.27%)	296 (10.83%)
Switzerland (N=1540)	0.07 (0.03, 0.14)	852 (55.32%)	570 (37.01%)	118 (7.66%)	0.07 (0.03, 0.14)	814 (55.79%)	542 (37.15%)	103 (7.06%)
USA (N=8350)	0.20 (0.13, 0.31)	1257 (15.05%)	4011 (48.04%)	3082 (36.91%)	0.21 (0.13, 0.33)	677 (12.78%)	2468 (46.60%)	2151 (40.62%)

FI, Frailty Index.

Additional Table 11. Associations of hobby engagement with Frailty Index by age, sex, marital status, education, residence area, smoking history, alcohol drinking history, weekly moderate to vigorous physical activity, labor force status, co-residence with children, and household wealth.

**Country**	**Age**				**Sex**			**Marital status**		
	**65-74 years**	**75-84 years**	**85 years**	***p***	**Male**	**Female**	***p***	**Married or partnered**	**Unmarried and others**	***p***
Austria	-0.025 (-0.029, -0.022)	-0.046 (-0.052, -0.041)	-0.117 (-0.126, -0.108)		-0.078 (-0.083, -0.073)	-0.056 (-0.060, -0.052)		-0.055 (-0.059, -0.051)	-0.069 (-0.074, -0.064)	
Belgium	-0.004 (-0.007, -0.001)	-0.023 (-0.026, -0.020)	-0.042 (-0.048,-0.037)		-0.025 (-0.028, -0.021)	-0.025 (-0.028, -0.022)		-0.019 (-0.022, -0.017)	-0.031 (-0.034, -0.027)	
China	-0.014 (-0.018, -0.011)	-0.013 (-0.019, -0.008)	0.053 (0.036, 0.070)		-0.008 (-0.012, -0.005)	-0.019 (-0.024, -0.014)		-0.011 (-0.014, -0.007)	-0.019 (-0.025, -0.013)	
Czech Republic	-0.030 (-0.032, -0.027)	-0.040 (-0.043, -0.037)	-0.092 (-0.099, -0.085)		-0.051 (-0.054, -0.048)	-0.052 (-0.054, -0.049)		-0.058 (-0.061, -0.056)	-0.043 (-0.046, -0.039)	
Denmark	0.003 (-0.001, 0.008)	-0.004 (-0.009, 0.001)	-0.057 (-0.066, -0.048)		-0.014 (-0.019, -0.010)	-0.070 (-0.075, -0.065)		-0.030 (-0.034, -0.025)	-0.048 (-0.053, -0.042)	
England	-0.008 (-0.010, -0.006)	-0.006 (-0.008, -0.004)	-0.027 (-0.031, -0.022)		-0.014 (-0.016, -0.012)	-0.010 (-0.012, -0.008)		-0.012 (-0.014, -0.010)	-0.010 (-0.012, -0.008)	
Estonia	-0.001 (-0.005, 0.002)	0.038 (-0.041, -0.036)	-0.066 (-0.071, -0.061)		-0.035 (-0.038, -0.031)	-0.050 (-0.052, -0.047)		-0.042 (-0.044, -0.039)	0.046 (-0.048, -0.043)	
France	-0.003 (-0.005, 0.000)	-0.003 (-0.005, 0.000)	-0.042 (-0.047, -0.037)		-0.028 (-0.030, -0.025)	-0.011 (-0.014, -0.009)		-0.018 (-0.020, -0.016)	-0.013 (-0.015, -0.010)	
Germany	-0.025 (-0.028, -0.021)	-0.033 (-0.037, -0.028)	-0.011 (-0.020, -0.002)		-0.031 (-0.035, -0.027)	-0.047 (-0.051, -0.043)		-0.037 (-0.040, -0.034)	-0.046 (-0.051, -0.040)	
Italy	0.004 (0.002, 0.006)	-0.020 (-0.022, -0.017)	-0.026 (-0.033, -0.019)		-0.007 (-0.009, -0.005)	-0.013 (-0.015, -0.010)		-0.006 (-0.008, -0.004)	-0.022 (-0.025, -0.018)	
Slovenia	-0.011 (-0.014, -0.008)	-0.026 (-0.029, -0.023)	-0.061 (-0.067, -0.054)		-0.036 (-0.039, -0.033)	-0.026 (-0.028, -0.023)		-0.028 (-0.030, -0.026)	-0.029 (-0.032, -0.025)	
Spain	0.003 (0.001, 0.005)	-0.022 (-0.024, -0.020)	-0.025 (-0.030, -0.019)		-0.020 (-0.022, -0.017)	-0.017 (-0.020, -0.015)		-0.013 (-0.015, -0.011)	-0.034 (-0.038, -0.030)	
Sweden	-0.018 (-0.023, -0.014)	-0.019 (-0.024, -0.015)	-0.082 (-0.090, -0.075)		-0.025 (-0.029, -0.021)	-0.062 (-0.067, -0.057)		-0.034 (-0.037, -0.030)	-0.053 (-0.058, -0.047)	
Switzerland	0.010 (0.007, 0.014)	-0.016 (-0.019, -0.012)	-0.031 (-0.039, -0.024)		-0.030 (-0.033, -0.026)	-0.004 (-0.008, 0.000)		-0.009 (-0.012, -0.006)	-0.036 (-0.041, -0.031)	
USA	-0.008 (-0.010, -0.006)	-0.016 (-0.018, -0.015)	-0.019 (-0.022, -0.016)		-0.013 (-0.014, -0.011)	-0.017 (-0.019, -0.016)		-0.012 (-0.013, -0.010)	-0.017 (-0.019, -0.016)	
Overall	-0.009 (-0.015, -0.002)	-0.022 (-0.028, -0.015)	-0.043 (-0.063, -0.023)	<0.001	-0.028 (-0.037, -0.018)	-0.032 (-0.043, -0.021)	0.566	-0.026 (-0.034, -0.017)	-0.034 (-0.043, -0.026)	0.153

Additional Table 10. (Continued).

Country	Education				Residence status			Co-residence with children		
	Primary	Secondary	Tertiary	*p*	Urban	Rural	*p*	Yes	No	*p*
Austria	-0.033<br>(-0.037, -0.028)	-0.083<br>(-0.089, -0.078)	-0.108<br>(-0.115, -0.100)		-0.100<br>(-0.105, -0.095)	-0.045<br>(-0.049, -0.041)		0.004<br>(-0.003, 0.011)	-0.080<br>(-0.083, -0.076)	
Belgium	-0.026<br>(-0.029, -0.023)	-0.010<br>(-0.015, -0.006)	-0.031<br>(-0.036, -0.026)		-0.030<br>(-0.032, -0.027)	-0.018<br>(-0.022, -0.015)		-0.030<br>(-0.037, -0.023)	-0.027<br>(-0.029, -0.025)	
China	-0.014<br>(-0.017, -0.011)	0.006<br>(-0.004, 0.017)	0.010<br>(0.002, 0.017)		-0.020<br>(-0.024, -0.016)	-0.004<br>(-0.008, 0.000)		-0.007<br>(-0.012, -0.003)	-0.015<br>(-0.018, -0.011)	
Czech Republic	-0.044<br>(-0.047, -0.041)	-0.059<br>(-0.062, -0.055)	-0.059<br>(-0.066, -0.052)		-0.054<br>(-0.056, -0.051)	-0.049<br>(-0.053, -0.046)		-0.069<br>(-0.075, -0.062)	-0.050<br>(-0.052, -0.048)	
Denmark	-0.015<br>(-0.022, -0.009)	-0.046<br>(-0.051, -0.041)	-0.043<br>(-0.052, -0.035)		-0.035<br>(-0.039, -0.031)	-0.059<br>(-0.067, -0.051)		0.005<br>(-0.026, 0.036)	-0.041<br>(-0.045, -0.038)	
England	-0.009<br>(-0.011, -0.007)	-0.014<br>(-0.016, -0.012)	-0.012<br>(-0.017, -0.006)		-	-		-0.013<br>(-0.018, -0.008)	0.011<br>(-0.013, -0.010)	
Estonia	-0.038<br>(-0.041, 0.036)	-0.048<br>(-0.051, -0.045)	-0.056<br>(-0.062, -0.050)		-0.047<br>(-0.049, -0.044)	-0.035<br>(-0.039, -0.032)		-0.041<br>(-0.043, -0.039)	-0.042<br>(-0.045, -0.040)	
France	-0.018<br>(-0.020, -0.015)	-0.017<br>(-0.021, -0.014)	-0.011<br>(-0.016, -0.005)		-0.016<br>(-0.019, -0.014)	-0.018<br>(-0.021, -0.015)		0.019<br>(0.013, 0.025)	-0.020<br>(-0.022, -0.019)	
Germany	-0.035<br>(-0.042, -0.029)	-0.050<br>(-0.053, -0.046)	0.000<br>(-0.006, 0.005)		-0.041<br>(-0.045, -0.037)	-0.026<br>(-0.031, -0.022)		-0.009<br>(-0.021, 0.003)	-0.041<br>(-0.044, -0.038)	
Italy	-0.011<br>(-0.013, -0.009)	0.003<br>(-0.001, 0.007)	-0.034<br>(-0.041, -0.027)		-0.009<br>(-0.011, -0.007)	-0.012<br>(-0.015, -0.010)		-0.012<br>(-0.016, -0.008)	-0.011<br>(-0.013, -0.009)	
Slovenia	-0.035<br>(-0.038, -0.032)	-0.023<br>(-0.025, -0.020)	-0.022<br>(-0.029, -0.016)		-0.029<br>(-0.031, -0.026)	-0.035<br>(-0.038, -0.033)		-0.057<br>(-0.061, -0.052)	-0.027<br>(-0.029, -0.025)	
Spain	-0.018<br>(-0.020, -0.016)	-0.039<br>(-0.048, -0.031)	-0.023<br>(-0.030, -0.017)		-0.024<br>(-0.026, -0.022)	0.009<br>(0.004, 0.014)		-0.026<br>(-0.030, -0.023)	-0.015<br>(-0.017, -0.013)	
Sweden	-0.047<br>(-0.051, -0.044)	-0.030<br>(-0.036, -0.024)	-0.034<br>(-0.043, -0.025)		-0.053<br>(-0.057, -0.050)	-0.030<br>(-0.035, -0.025)		-0.019<br>(-0.030, -0.007)	-0.042<br>(-0.045, -0.039)	
Switzerland	-0.030<br>(-0.034, -0.025)	-0.002<br>(-0.006, 0.001)	-0.029<br>(-0.037, -0.020)		-0.014<br>(-0.018, -0.020)	-0.021<br>(-0.025, -0.018)		-0.007<br>(-0.016, -0.002)	-0.015<br>(-0.018, -0.012)	
USA	-0.013<br>(-0.016, -0.011)	-0.016<br>(-0.017, -0.015)	-0.013<br>(-0.015, -0.011)		-0.015<br>(-0.017, -0.014)	-0.011<br>(-0.013, -0.009)		-0.011<br>(-0.013, -0.009)	-0.014<br>(-0.015, -0.012)	
Overall	-0.026<br>(-0.032, -0.019)	-0.029<br>(-0.041, -0.016)	-0.031<br>(-0.045, -0.017)	<0.001	-0.035<br>(-0.047, -0.022)	-0.025<br>(-0.035, -0.016)	0.239	-0.019<br>(-0.031, -0.007)	-0.030<br>(-0.040, -0.020)	0.149

Additional Table 10. (Continued).

Country	Smoking history				Alcohol drinking history				Weekly moderate to vigorous physical activity		
	**Never smoker**	**Former smoker**	**Current smoker**	***p***	**Never drinker**	**Former drinker**	**Current drinker**	***p***	**Physical activity**	**Physical inactivity**	***p***
Austria	-0.061 (-0.064, -0.057)	-0.096 (-0.105, -0.087)	-0.050 (-0.061, -0.039)		-0.056 (-0.061, -0.039)	-0.033 (-0.045, -0.022)	-0.040 (-0.045, -0.036)		-0.026 (-0.031, -0.021)	-0.061 (-0.065, -0.057)	
Belgium	-0.027 (-0.029, -0.024)	-0.014 (-0.018, -0.009)	-0.012 (-0.020, -0.005)		-0.019 (-0.023, -0.016)	-0.026 (-0.039, -0.014)	-0.021 (-0.024, -0.019)		0.002 (-0.001, 0.006)	-0.030 (-0.032, -0.027)	
China	-0.010 (-0.014, -0.006)	-0.001 (-0.007, 0.005)	-0.008 (-0.013, -0.003)		-0.013 (-0.018, -0.009)	-0.024 (-0.031, -0.017)	0.001 (-0.003, 0.006)		-0.006 (-0.012, 0.000)	-0.014 (-0.017, -0.011)	
Czech Republic	-0.059 (-0.062, -0.057)	-0.029 (-0.034, -0.024)	-0.014 (-0.020, -0.008)		-0.055 (-0.058, -0.052)	-0.040 (-0.047, -0.033)	-0.036 (-0.039, -0.033)		-0.026 (-0.029, -0.022)	-0.053 (-0.056, -0.051)	
Denmark	-0.066 (-0.071, -0.061)	-0.020 (-0.026, -0.014)	-0.043 (-0.051, -0.035)		-0.049 (-0.056, -0.042)	-0.103 (-0.126, -0.079)	-0.036 (-0.040, -0.032)		-0.038 (-0.043, -0.032)	-0.033 (-0.038, -0.029)	
England	-0.017 (-0.019, -0.014)	-0.009 (-0.011, -0.007)	0.003 (-0.002, 0.008)		-0.014 (-0.017, -0.012)	-0.009 (-0.011, -0.007)	0.003 (-0.002, 0.008)		-0.005 (-0.006, -0.003)	-0.014 (-0.017, -0.012)	
Estonia	-0.044 (-0.046, -0.041)	-0.056 (-0.061, -0.050)	-0.023 (-0.030, -0.015)		-0.050 (-0.052, -0.048)	-0.023 (-0.030, -0.016)	-0.020 (-0.025, -0.014)		-0.019 (-0.022, -0.016)	-0.047 (-0.049, -0.044)	
France	-0.019 (-0.021, -0.016)	-0.023 (-0.027, -0.018)	-0.010 (-0.016, -0.003)		-0.025 (-0.028, -0.022)	-0.139 (-0.150, -0.127)	-0.012 (-0.014, -0.009)		-0.003 (-0.006, 0.000)	-0.021 (-0.024, -0.019)	
Germany	-0.038 (-0.041, -0.035)	-0.026 (-0.033, -0.019)	-0.055 (-0.063, -0.047)		-0.056 (-0.059, -0.052)	-0.146 (-0.169, -0.123)	-0.012 (-0.017, -0.008)		-0.006 (-0.010, -0.001)	-0.042 (-0.045, -0.038)	
Italy	-0.011 (-0.013, -0.009)	-0.013 (-0.016, -0.009)	0.012 (0.007, 0.018)		-0.013 (-0.016, -0.011)	-0.012 (-0.025, 0.001)	-0.004 (-0.007, -0.002)		0.000 (-0.003, 0.002)	-0.013 (-0.015, -0.010)	
Slovenia	-0.029 (-0.031, -0.027)	-0.041 (-0.046, -0.035)	-0.018 (-0.027, -0.009)		-0.034 (-0.037, -0.031)	0.000 (-0.009, 0.008)	-0.016 (-0.019, -0.013)		-0.016 (-0.019, -0.013)	-0.029 (-0.031, -0.026)	
Spain	-0.019 (-0.021, -0.017)	-0.015 (-0.019, -0.011)	0.013 (0.007, 0.020)		-0.024 (-0.026, -0.021)	-0.026 (-0.046, -0.006)	0.003 (0.001, 0.006)		-0.007 (-0.009, -0.004)	-0.023 (-0.025, -0.021)	
Sweden	-0.037 (-0.041, -0.033)	-0.056 (-0.062, -0.051)	-0.005 (-0.016, 0.005)		-0.059 (-0.063, -0.054)	-0.057 (-0.064, -0.050)	-0.020 (-0.024, -0.015)		-0.008 (-0.013, -0.004)	-0.054 (-0.058, -0.050)	
Switzerland	-0.011 (-0.014, -0.008)	-0.047 (-0.055, -0.040)	-0.022 (-0.029, -0.015)		-0.008 (-0.013, -0.003)	0.027 (0.004, 0.050)	-0.025 (-0.028, -0.021)		0.009 (0.005, 0.013)	-0.020 (-0.023, -0.016)	
USA	-0.015 (-0.017, -0.013)	-0.015 (-0.016, -0.013)	-0.005 (-0.009, -0.001)		-0.016 (-0.017, -0.014)	-0.001 (-0.004, 0.002)	-0.014 (-0.015, -0.012)		-0.007 (-0.008, -0.005)	-0.016 (-0.018, -0.014)	
Overall	-0.031 (-0.040, -0.021)	-0.031 (-0.043, -0.018)	-0.016 (-0.026, -0.005)	0.046	-0.033 (-0.042, -0.023)	-0.041 (-0.066, -0.015)	-0.017 (-0.024, -0.010)	0.024	-0.010 (-0.016, -0.004)	-0.031 (-0.039, -0.023)	<0.001

Additional Table 10. (Continued).

Country	Labor force status			Household wealth			
	Currently not working	Currently working	*p*	Low level	Middle level	High level	*p*
Austria	-0.065 (-0.068, -0.062)	-0.063 (-0.066, -0.060)		-0.060 (-0.065, -0.055)	-0.057 (-0.062, -0.053)	-0.066 (-0.075, -0.057)	
Belgium	-0.025 (-0.027, -0.023)	-0.025 (-0.027, -0.023)		-0.019 (-0.023, -0.014)	-0.017 (-0.020, -0.014)	-0.016 (-0.019, -0.012)	
China	-0.014 (-0.018, -0.010)	-0.012 (-0.015, -0.009)		-0.031 (-0.038, -0.025)	-0.011 (-0.015, -0.007)	-0.009 (-0.013, -0.004)	
Czech Republic	-0.052 (-0.054, -0.050)	-0.051 (-0.053, -0.049)		-0.045 (-0.048, -0.042)	-0.058 (-0.061, -0.055)	-0.019 (-0.026, -0.012)	
Denmark	-0.040 (-0.044, -0.036)	-0.041 (-0.045, -0.038)		-0.022 (-0.030, -0.015)	-0.056 (-0.062, -0.049)	-0.032 (-0.037, -0.027)	
England	-0.011 (-0.012, -0.009)	-0.011 (-0.013, -0.010)		-0.005 (-0.007, -0.003)	-0.010 (-0.012, -0.008)	-0.011 (-0.014, -0.008)	
Estonia	-0.045 (-0.047, -0.043)	-0.045 (-0.047, -0.043)		-0.033 (-0.036, -0.029)	-0.071 (-0.079, -0.062)	-0.032 (-0.037 -0.027)	
France	-0.019 (-0.021, -0.017)	-0.018 (-0.020, -0.016)		-0.029 (-0.033, -0.025)	-0.006 (-0.009, -0.003)	-0.008 (-0.011, -0.005)	
Germany	-0.037 (-0.040, -0.035)	-0.039 (-0.042, -0.036)		-0.037 (-0.043, -0.031)	-0.031 (-0.035, -0.027)	-0.043 (-0.049, -0.037)	
Italy	-0.010 (-0.012, -0.009)	-0.010 (-0.011, -0.008)		-0.019 (-0.022, -0.016)	-0.008 (-0.010, -0.006)	-0.001 (-0.004, 0.003)	
Slovenia	-0.030 (-0.032, -0.028)	-0.030 (-0.032, -0.028)		-0.029 (-0.032, -0.026)	-0.029 (-0.032, -0.026)	-0.031 (-0.037, -0.026)	
Spain	-0.019 (-0.021, -0.018)	-0.019 (-0.021, -0.017)		-0.023 (-0.027, -0.020)	-0.012 (-0.014, -0.009)	-0.021 (-0.025, -0.018)	
Sweden	-0.048 (-0.051, -0.044)	-0.041 (-0.044, -0.038)		-0.081 (-0.088, -0.074)	-0.039 (-0.044, -0.034)	-0.020 (-0.025, -0.016)	
Switzerland	-0.020 (-0.023, -0.017)	-0.016 (-0.019, -0.013)		-0.026 (-0.034, -0.019)	-0.015 (-0.019, -0.010)	-0.019 (-0.023, -0.016)	
USA	-0.017 (-0.018, -0.016)	-0.015 (-0.016, -0.014)		-0.021 (-0.023, -0.018)	-0.008 (-0.010, -0.007)	-0.018 (-0.019, -0.016)	
Overall	-0.030 (-0.039, -0.022)	-0.029 (-0.038, -0.021)	0.869	-0.032 (-0.041, -0.023)	-0.028 (-0.040, -0.017)	-0.023 (-0.031, -0.015)	<0.001

CHARLS, China Health and Retirement Longitudinal Study; ELSA, English Longitudinal Study of Ageing; HRS, Health and Retirement Study; SHARE, Survey of Health, Ageing and Retirement in Europe.

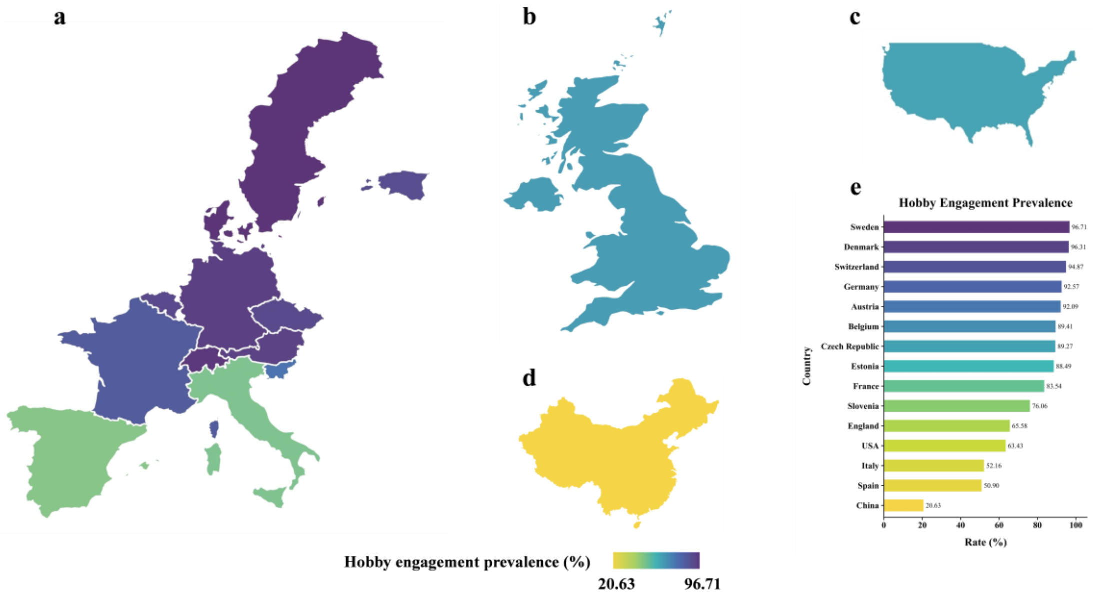

Additional Fig.1 Levels of hobby engagement prevalence among adults aged 65 and above across 15 countries. Panel A. China Health and Retirement Longitudinal Study. **Panel B.** English Longitudinal Study on Ageing. **Panel C.** Health and Retirement Study. **Panel D.** Survey of Health, Ageing and Retirement in Europe. Panel E. Prevalence of hobby engagement.

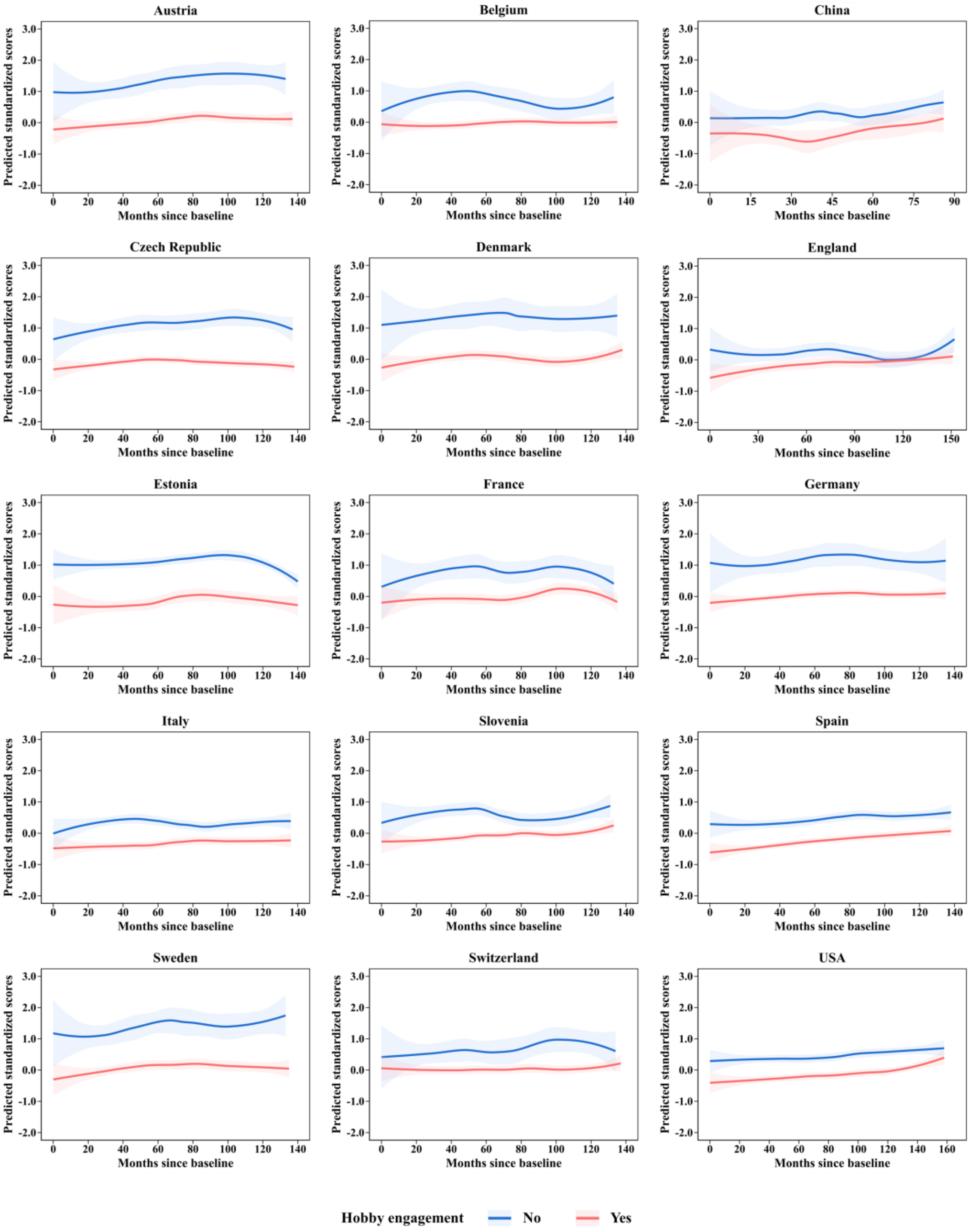
Austria
Belgium
China
Czech Republic
Denmark
England
Estonia
France
Germany
Italy
Slovenia
Spain
Sweden
Switzerland
USA
Predicted standardized scores
Months since baseline
Hobby engagement
No
Yes

Additional Fig.2 Predicted standardized scores for Frailty Index over time by baseline hobby engagement. Data were presented as the average adjusted predictions with 95% CIs by hobby engagement and months since baseline. CI, confidence interval.

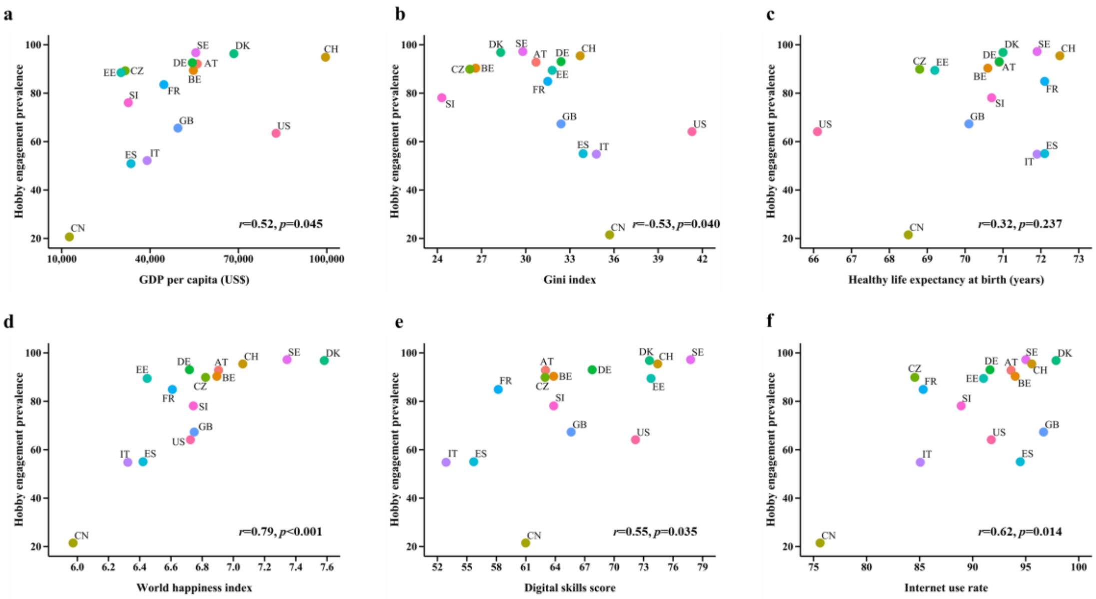

Additional Fig.3 Pearson correlations between hobby engagement prevalence and country-level factors. (a), GDP per capita; (b), Gini index; (c), Healthy life expectancy at birth; (d), World happiness index; (e), Digital skills score; (f) Internet use rate. GDP, gross domestic product; AT, Austria; BE, Belgium; CN, China; CZ, Czech Republic; DK, Denmark; EN, England; EE, Estonia; FR, France; DE, Germany; IT, Italy; SI, Slovenia; ES, Spain; SE, Sweden; CH, Switzerland; US, United States.

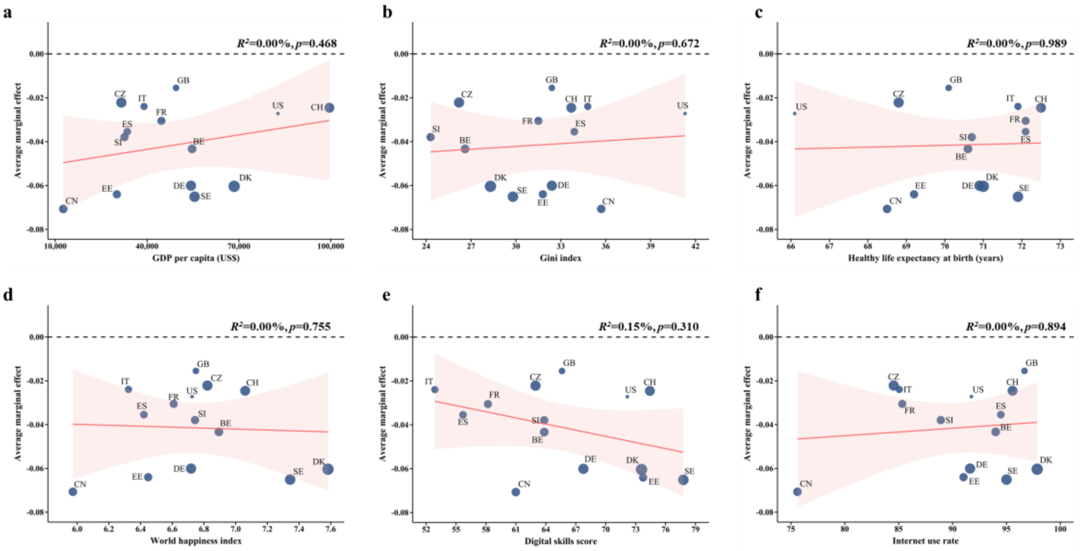

Additional Fig.4 Bubble plot with fitted meta regression line of the average marginal effect for Frailty Index and country-level factors. (a), GDP per capita; (b), Gini index; (c), Healthy life expectancy at birth; (d), World happiness index; (e), Digital skills score; (f) Internet use rate. Data are presented as the average marginal effects (bubbles), 95% confidence intervals (shaded area), and the linear prediction (red line). The R^2 quantifies the proportion of heterogeneity that can be accounted for by each country-level factor. The p indicates whether the average marginal effect was associated with each country-level factor. GDP, gross domestic product; AT, Austria; BE, Belgium; CN, China; CZ, Czech Republic; DK, Denmark; EN, England; EE, Estonia; FR, France; DE, Germany; IT, Italy; SI, Slovenia; ES, Spain; SE, Sweden; CH, Switzerland; US, United States.

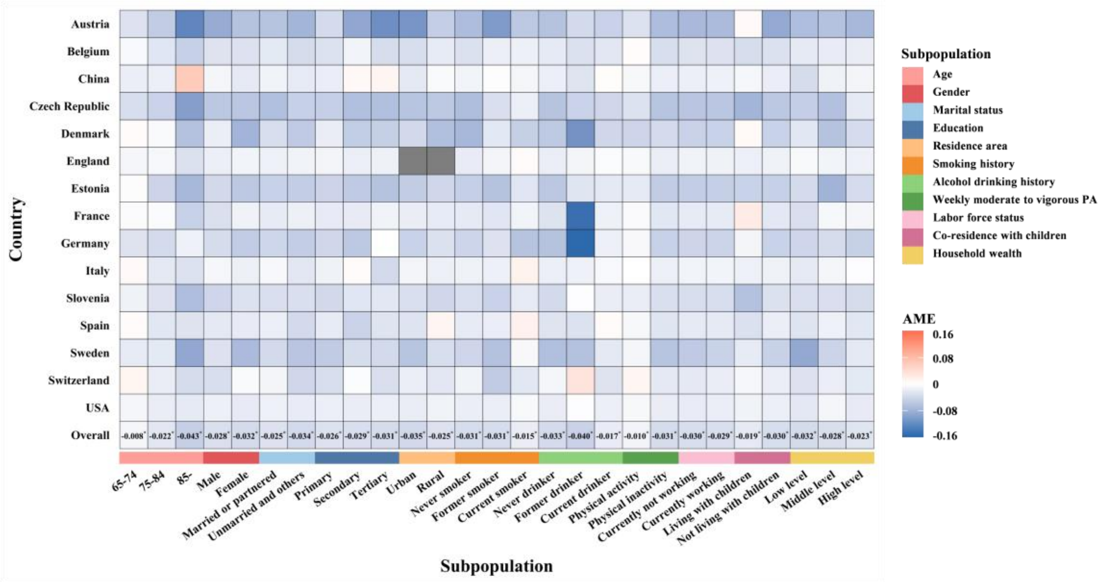

Additional Fig.5 Heatmap of association between hobby engagement and the Frailty Index across subpopulations in different countries. Each cell represents the covariate-adjusted AME of a specific subpopulation characteristic in a given country, with colour intensity indicating effect size (blue = negative, red = positive; see colour scale). Grey cells indicate missing data on the residence area in England. Subpopulations are grouped by demographic, socioeconomic, health behaviour, and living condition factors, as indicated in the legend. The overall row shows pooled AMEs across all countries. AME – average marginal effect, PA – physical activity.

Additional Results of Sensitivity Analysis

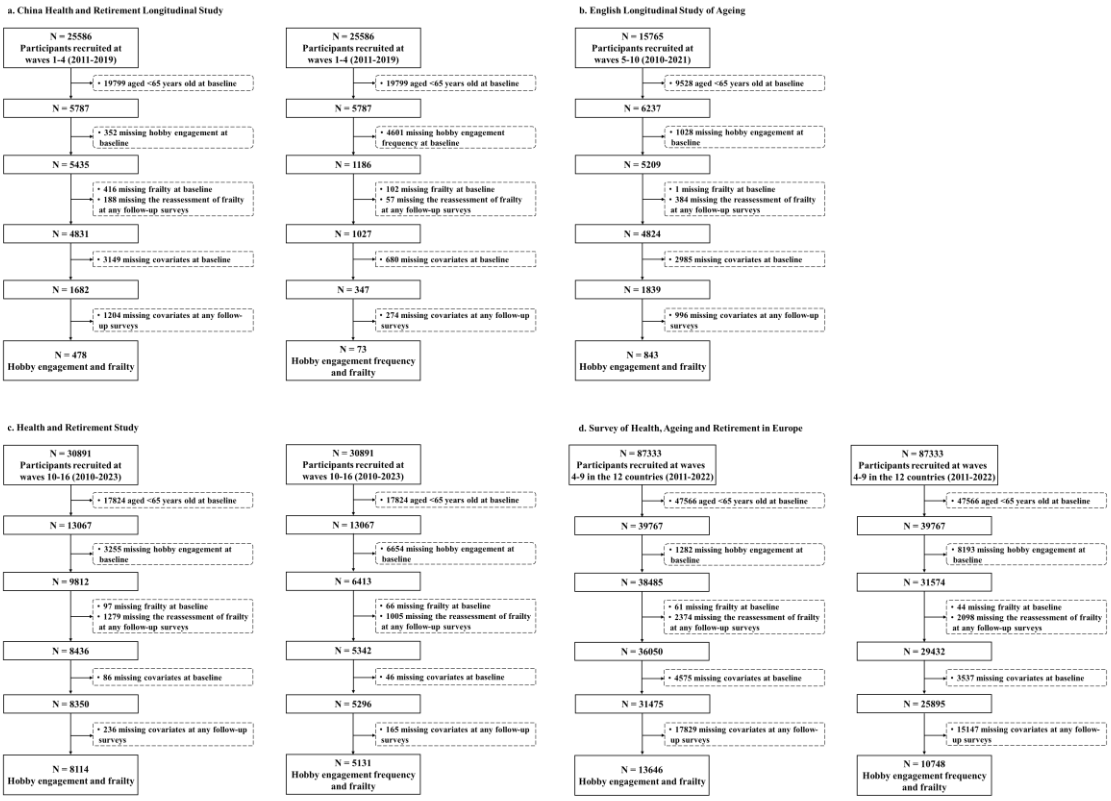

Additional Fig.6 Flowchart of sample selection for analysis using complete cases. (a), China Health and Retirement Longitudinal Study (CHARLS); (b), English Longitudinal Study on Ageing (ELSA); (c) Health and Retirement Study (HRS); (d) Survey of Health, Ageing and Retirement in Europe (SHARE).

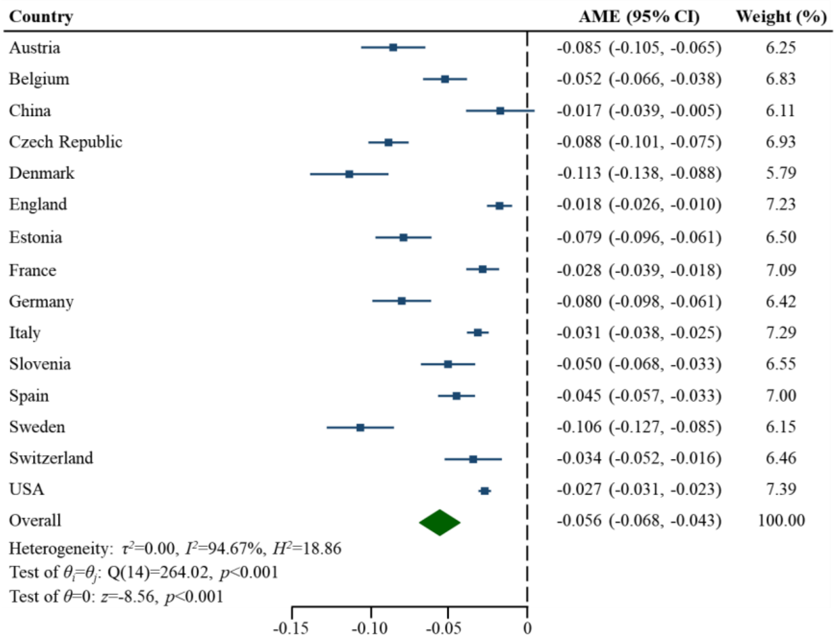

Additional Fig.7 Association between hobby engagement and Frailty Index using complete cases (n=23081 from 15 countries). AME, average marginal effect.

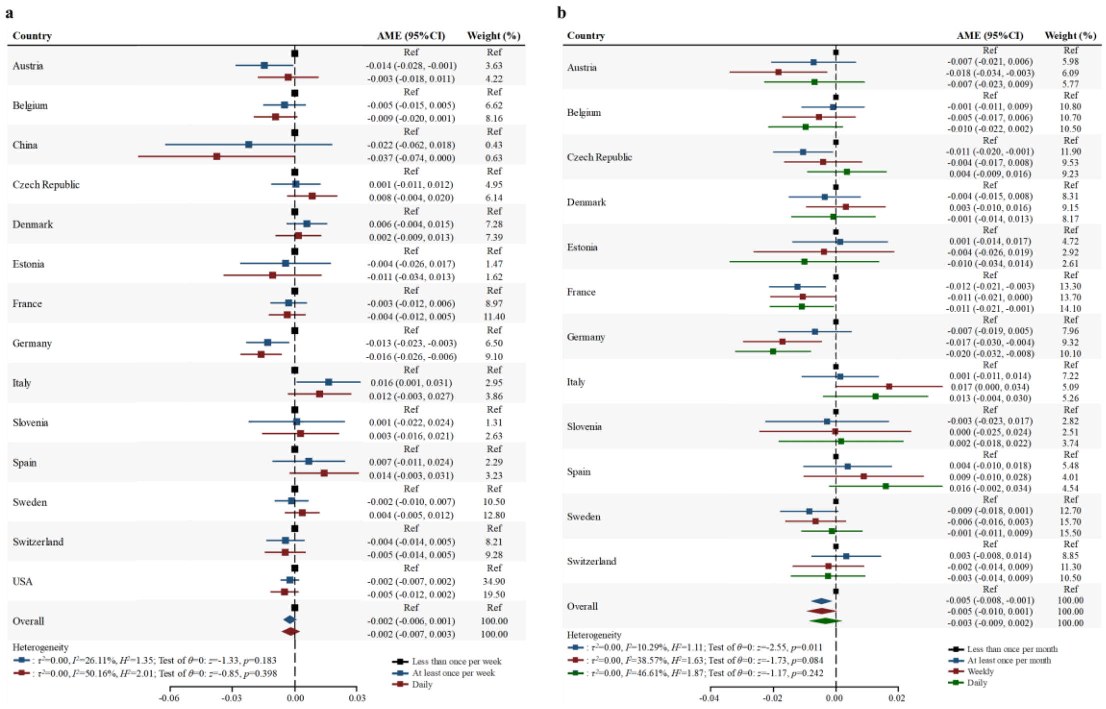

Additional Fig.8 Association between hobby engagement frequency and Frailty Index using complete cases. (a) Results from 14 countries using three frequency categories: less than once per week, at least once per week, and daily (*n*=15952); (b) Results from 12 countries using four frequency categories: less than once per month, at least once per month, weekly, and daily (*n*=10748). AME, average marginal effect.

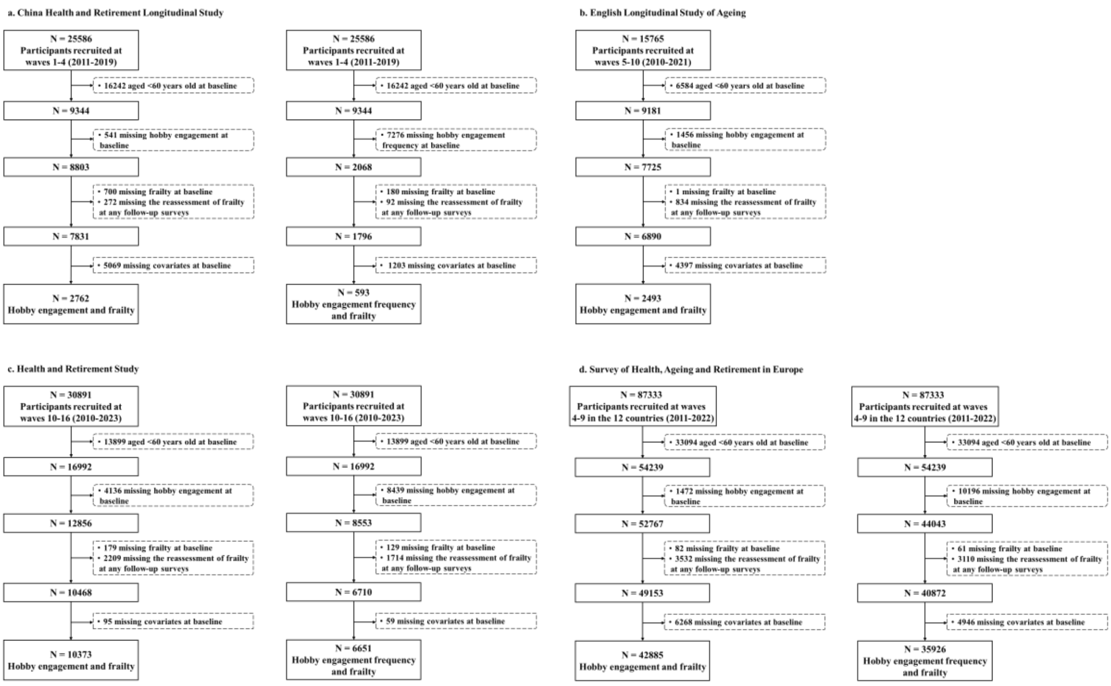

Additional Fig.9 Flowchart of sample selection for analysis among adults aged 60 and above. (a), China Health and Retirement Longitudinal Study (CHARLS); (b), English Longitudinal Study on Ageing (ELSA); (c) Health and Retirement Study (HRS); (d) Survey of Health, Ageing and Retirement in Europe (SHARE).

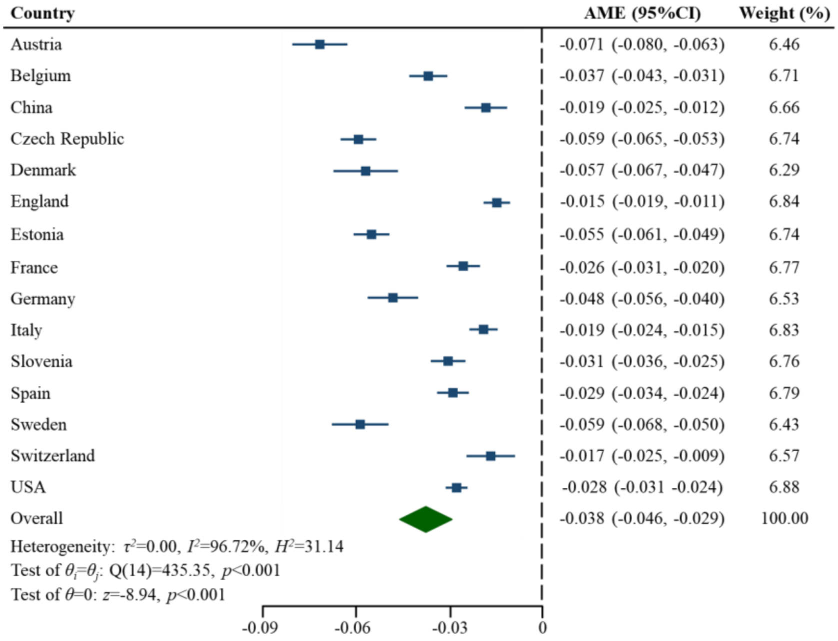

Additional Fig.10 Association between hobby engagement and Frailty Index among adults aged 60 and above (n=58513 from 15 countries). AME, average marginal effect.

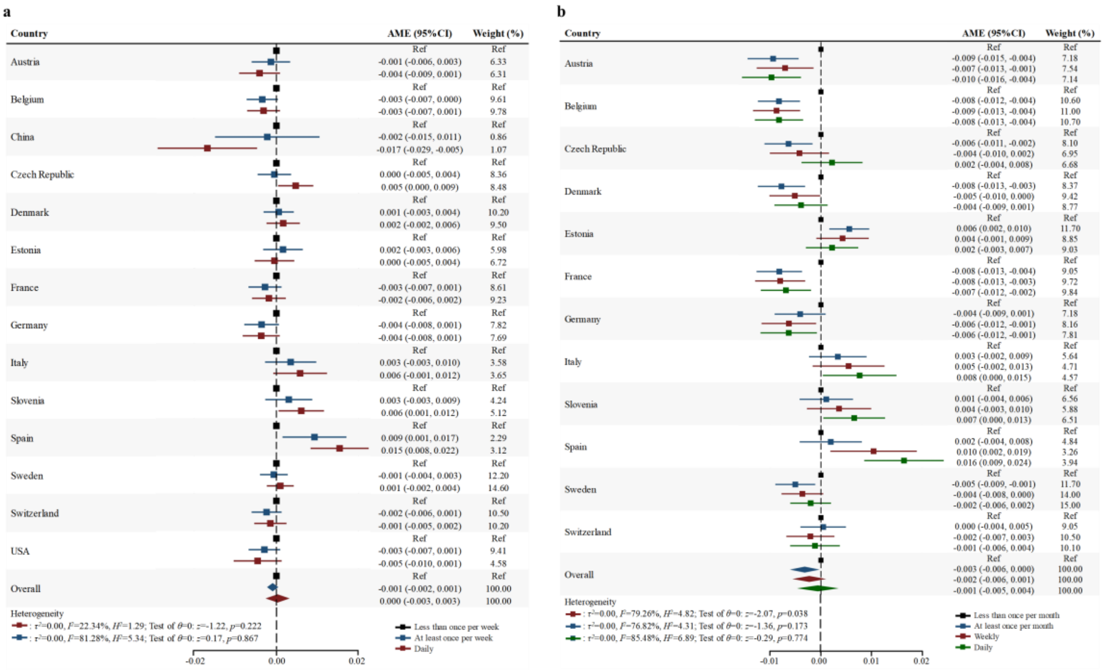

Additional Fig.11 Association between hobby engagement frequency and Frailty Index among adults aged 60 and above. (a) Results from 14 countries using three frequency categories: less than once per week, at least once per week, and daily (*n*=43170); (b) Results from 12 countries using four frequency categories: less than once per month, at least once per month, weekly, and daily (*n*=35926). AME, average marginal effect.

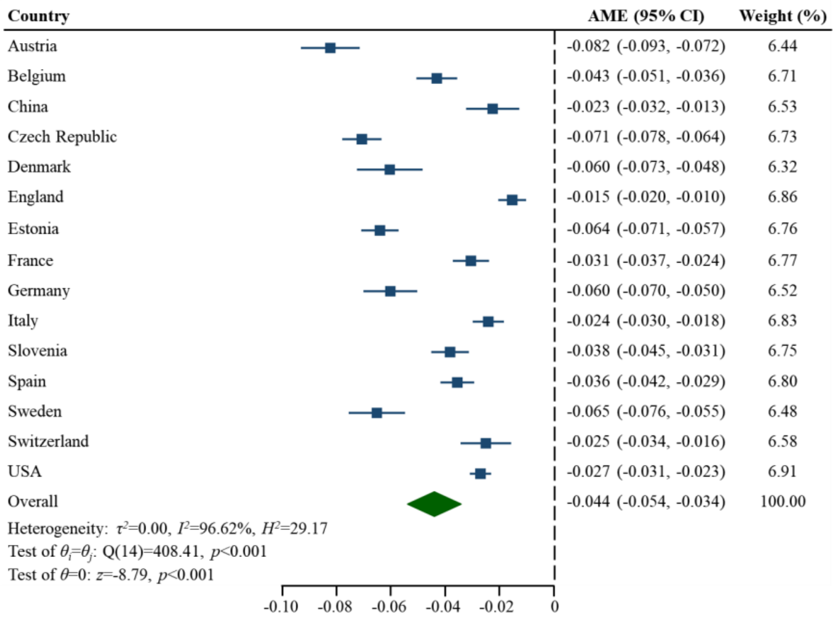

Additional Fig.12 Association between hobby engagement and Frailty Index using the inverse probability weights of attrition (n=43346 from 15 countries). AME, average marginal effect.

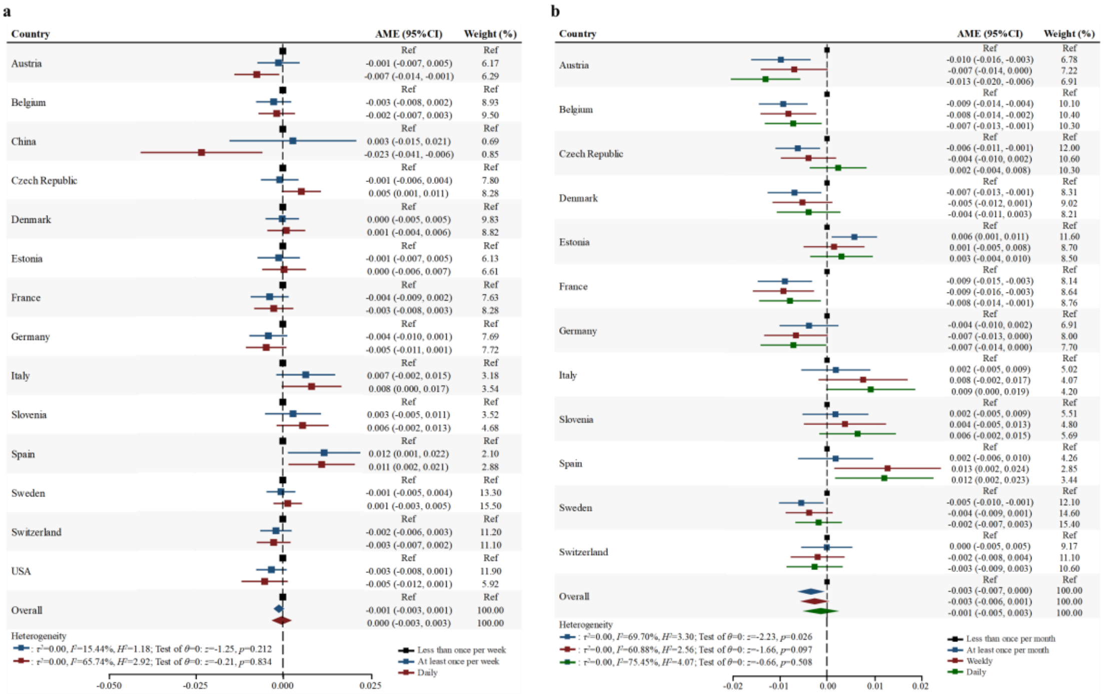

Additional Fig.13 Association between hobby engagement frequency and Frailty Index using the inverse probability weights of attrition. (a) Results from 14 countries using three frequency categories: less than once per week, at least once per week, and daily (n=31538); (b) Results from 12 countries using four frequency categories: less than once per month, at least once per month, weekly, and daily (n=25895). AME, average marginal effect.

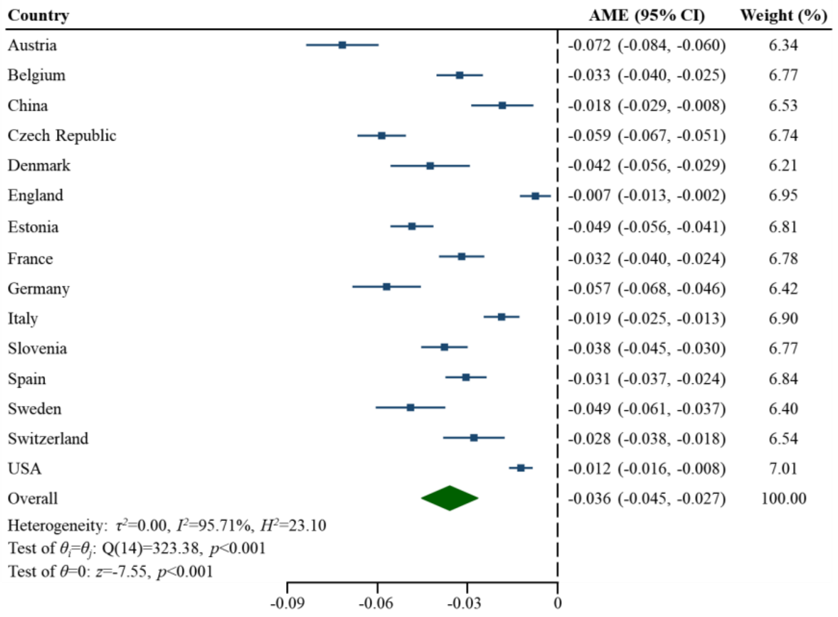

Additional Fig.14 Association between hobby engagement and Frailty Index using lagged models (*n*=43346 from 15 countries). AME, average marginal effect.

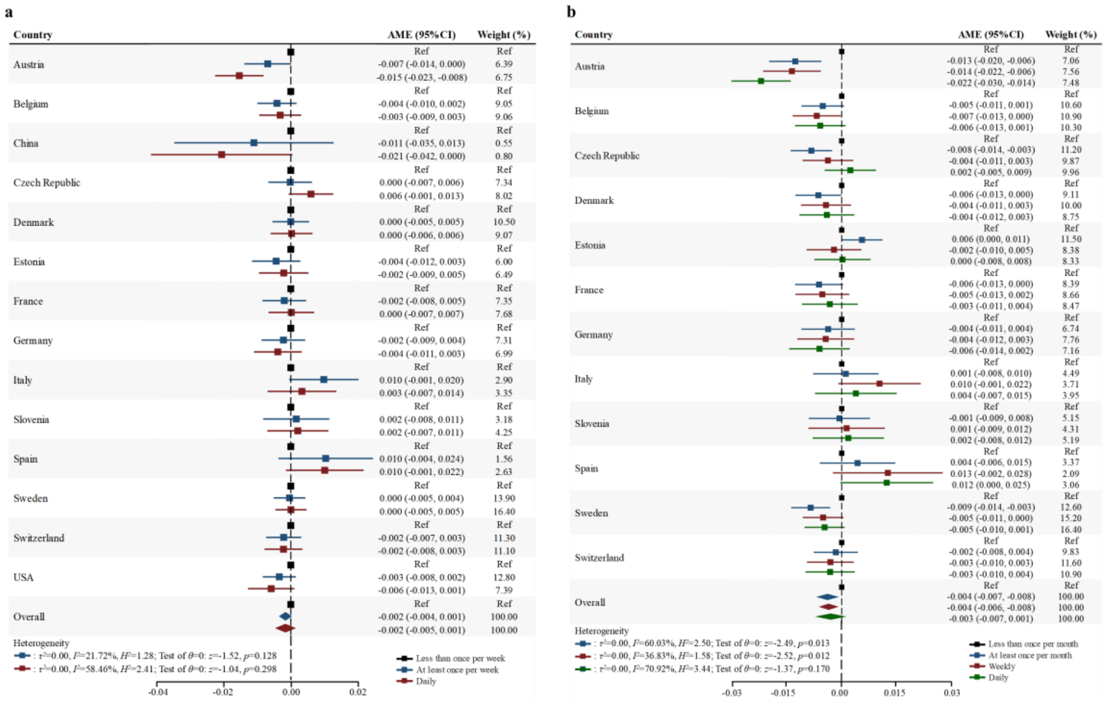

Additional Fig.15 Association between hobby engagement frequency and Frailty Index using lagged models. (a) Results from 14 countries using three frequency categories: less than once per week, at least once per week, and daily (*n*=31538); (b) Results from 12 countries using four frequency categories: less than once per month, at least once per month, weekly, and daily (*n*=25895). AME, average marginal effect.

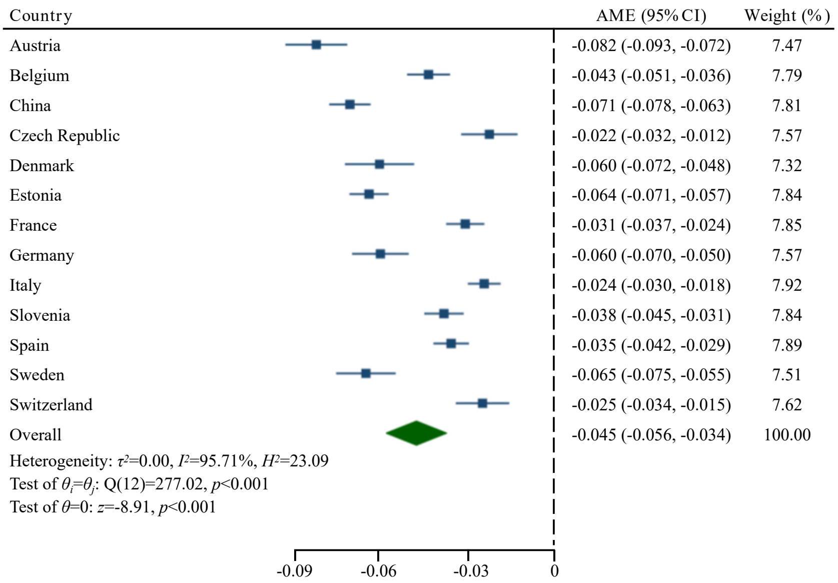

Additional Fig.16 Association between hobby engagement and Frailty Index excluding data from ELSA and HRS (n=34996 from 14 countries). AME, average marginal effect.

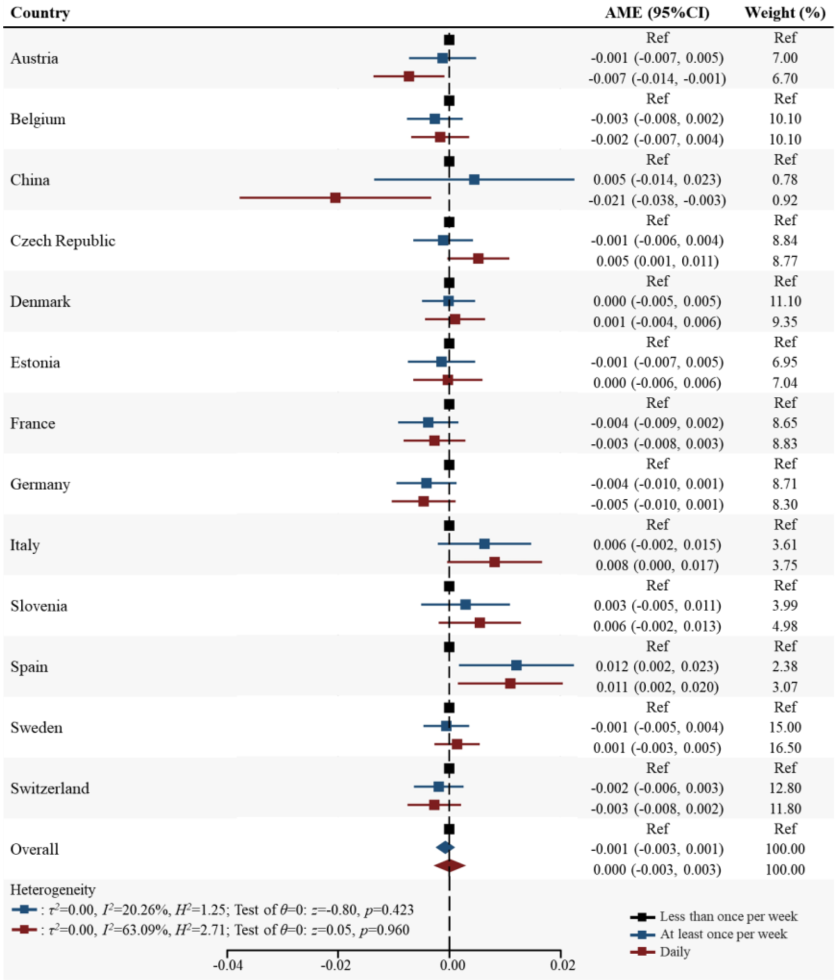

Additional Fig.17 Association between hobby engagement frequency and Frailty Index excluding data from ELSA and HRS using three frequency categories: less than once per week, at least once per week, and daily (n=26242 from 13 countries). AME, average marginal effect.

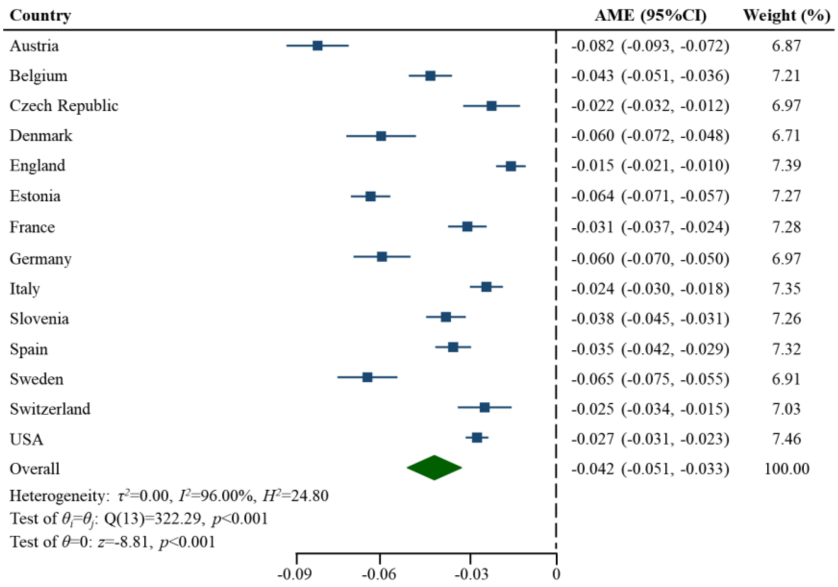

Additional Fig.18 Association between hobby engagement and Frailty Index excluding data from CHARLS (n=41664 from 14 countries). AME, average marginal effect.

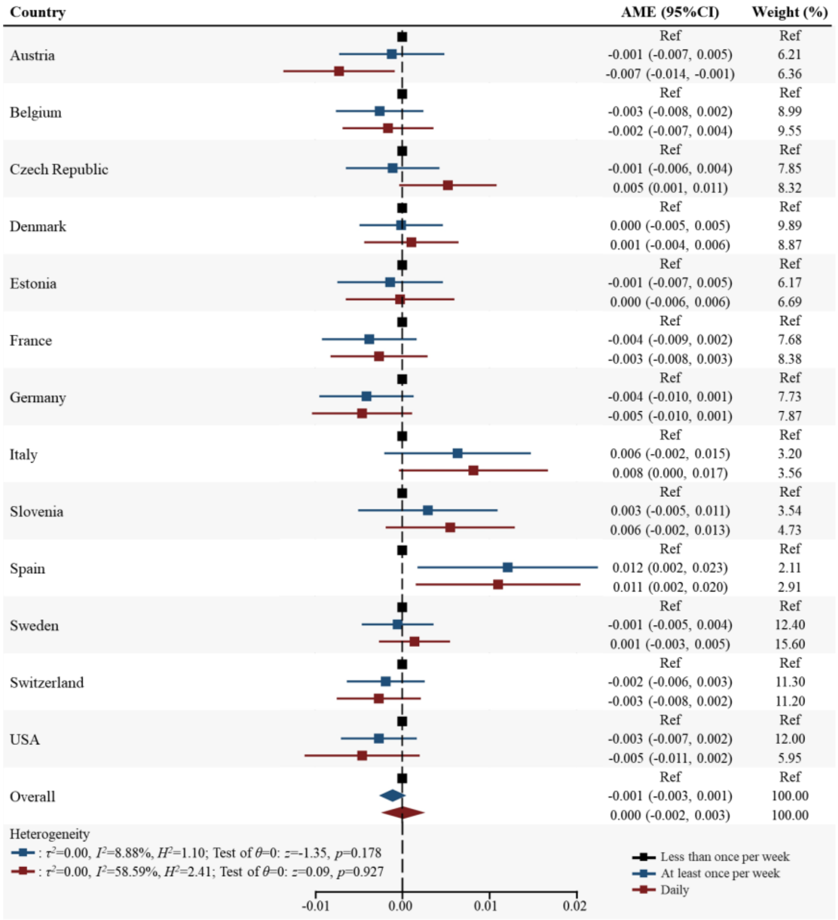

Additional Fig.19 Association between hobby engagement frequency and Frailty Index excluding data from CHARLS using three frequency categories: less than once per week, at least once per week, and daily (n=31191 from 13 countries). AME, average marginal effect.

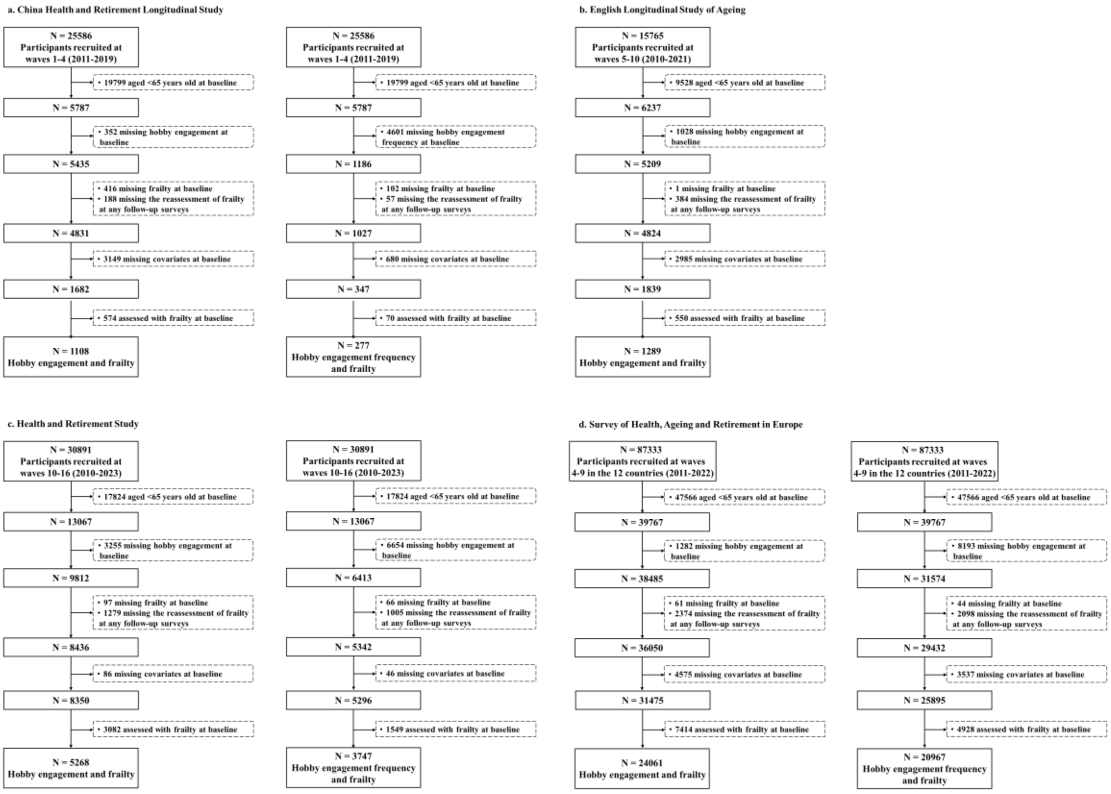

Additional Fig.20 Flowchart of sample selection for analysis regarding incident frailty using the COX regression model. (a), China Health and Retirement Longitudinal Study (CHARLS); (b), English Longitudinal Study on Ageing (ELSA); (c) Health and Retirement Study (HRS); (d) Survey of Health, Ageing and Retirement in Europe (SHARE).

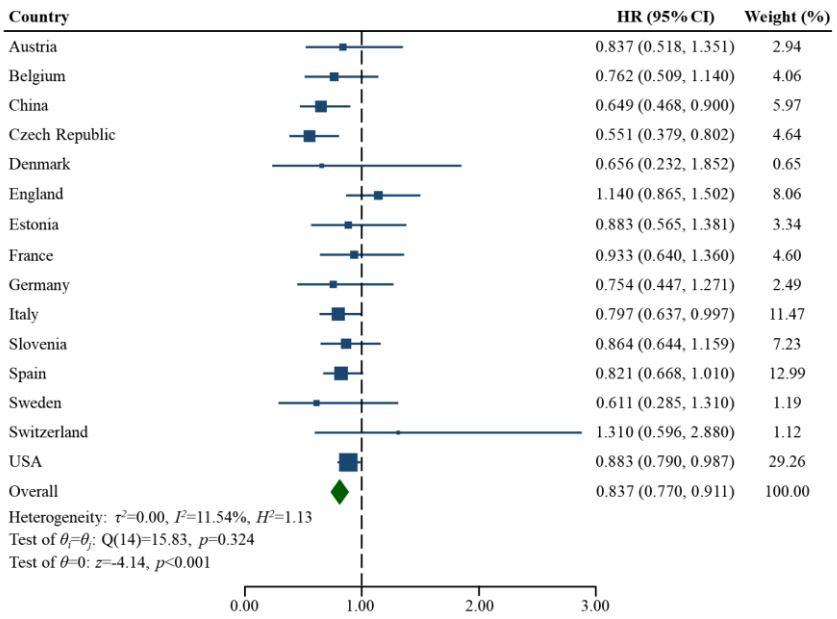

Additional Fig.21 Association between hobby engagement and incident frailty (*n*=31726 from 15 countries). HR, hazard ratio.

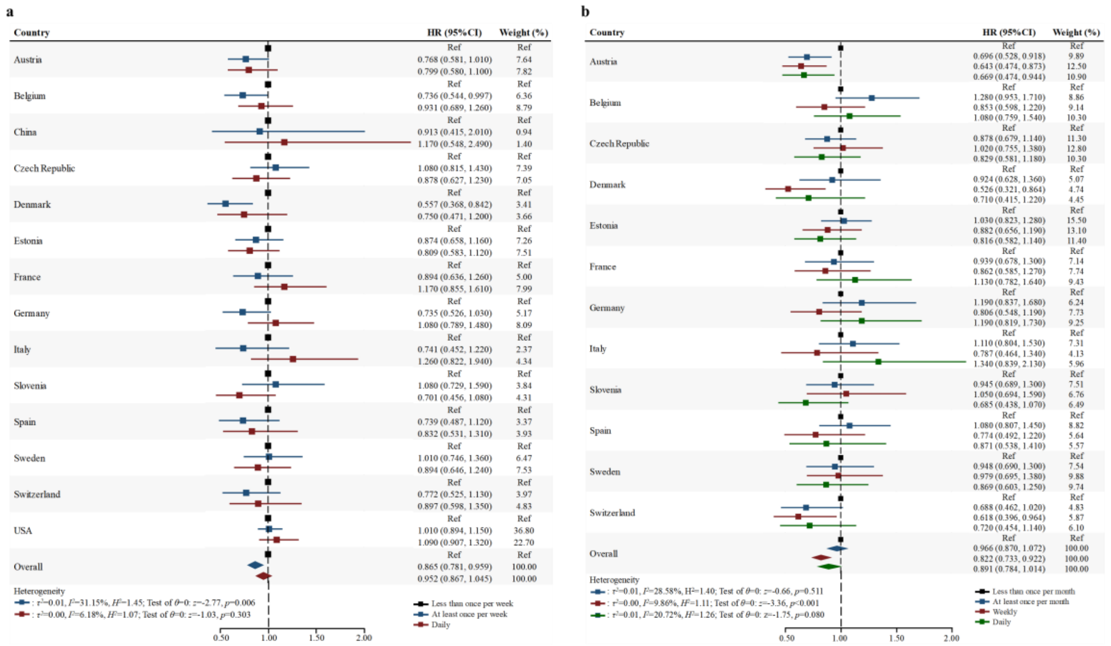

Additional Fig.22 Association between hobby engagement frequency and incident frailty. (a) Results from 14 countries using three frequency categories: less than once per week, at least once per week, and daily (n=24991); (b) Results from 12 countries using four frequency categories: less than once per month, at least once per month, weekly, and daily (n=20967). HR, hazard ratio.

Text S1. Explanation of the authorship change statement

YQ was added as a co-author after the submission of this manuscript due to his support in writing & editing the code needed to address peer reviewers' comments and expand existing analyses. To support this change, the authors have provided an authorship change form, co-signed by all authors; a new authorship contribution statement reflecting the authors' contribution to the work; and a new disclosure of interest statement. They also provided analytical code which the author contributed, including the code for the Cox model, the inverse probability weighting, the lagged panel analysis, main analysis of hobby engagement, and subpopulation effect estimates. These changes accurately reflect the revisions requested by the authors and implemented in the post-peer review version of the manuscript.
